# Quantitative vessel tortuosity: A potential CT imaging biomarker for distinguishing lung granulomas from adenocarcinomas

**DOI:** 10.1038/s41598-018-33473-0

**Published:** 2018-10-16

**Authors:** Mehdi Alilou, Mahdi Orooji, Niha Beig, Prateek Prasanna, Prabhakar Rajiah, Christopher Donatelli, Vamsidhar Velcheti, Sagar Rakshit, Michael Yang, Frank Jacono, Robert Gilkeson, Philip Linden, Anant Madabhushi

**Affiliations:** 1 Department of Biomedical Engineering, Case Western Reserve University, Cleveland, OH 44106 USA; 2 University Hospital Case Medical Center, Cleveland, OH 44106 USA; 3 Taussig Cancer Institute, Cleveland Clinic, Cleveland, OH 44106 USA; 4 Louis Stokes Cleveland VA Medical Center, Cleveland, OH 44106 USA

**Keywords:** Biomedical engineering, Computer science

## Abstract

Adenocarcinomas and active granulomas can both have a spiculated appearance on computed tomography (CT) and both are often fluorodeoxyglucose (FDG) avid on positron emission tomography (PET) scan, making them difficult to distinguish. Consequently, patients with benign granulomas are often subjected to invasive surgical biopsies or resections. In this study, quantitative vessel tortuosity (QVT), a novel CT imaging biomarker to distinguish between benign granulomas and adenocarcinomas on routine non-contrast lung CT scans is introduced. Our study comprised of CT scans of 290 patients from two different institutions, one cohort for training (N = 145) and the other (N = 145) for independent validation. In conjunction with a machine learning classifier, the top informative and stable QVT features yielded an area under receiver operating characteristic curve (ROC AUC) of 0.85 in the independent validation set. On the same cohort, the corresponding AUCs for two human experts including a radiologist and a pulmonologist were found to be 0.61 and 0.60, respectively. QVT features also outperformed well known shape and textural radiomic features which had a maximum AUC of 0.73 (p-value = 0.002), as well as features learned using a convolutional neural network AUC = 0.76 (p-value = 0.028). Our results suggest that QVT features could potentially serve as a non-invasive imaging biomarker to distinguish granulomas from adenocarcinomas on non-contrast CT scans.

## Introduction

Histoplasmosis is among the most common endemic fungal infection in the United States^1^. On chest CT, active fungal infection induced granulomas and malignant nodules may both have a suspicious spiculated appearance. Moreover, if the fungal infection is active, both lesions may appear ‘hot’ on a PET scan. On screening CT scans, the great majority of pulmonary nodules are benign (95%) and a significant proportion represent granulomas caused by a fungal infection^2^. A number of these patients identified with lung nodules end up having to undergo a surgical intervention in the form of a biopsy, bronchoscopy or a surgical wedge resection for histopathologic confirmation of presence or absence of a malignancy^3^. Even if no biopsy is recommended, the majority of these patients will have to undergo repeat CT scans for continued assessment of the nodule, thereby exposing them to unnecessary and potentially harmful radiation. Over 1 million people in the US are annually subjected to a CT guided or bronchoscopic biopsy and over 60,000^4^ are subjected to a surgical wedge resection for pathologic confirmation of a pulmonary nodule found on a CT scan^5^. Approximately 30% of pulmonary nodules identified as suspicious on a CT scan and that are subsequently biopsied or resected will finally be confirmed on histopathology to be benign.

Recognizing that there is an unmet need for decision support tools for analysis and interpretation of chest CT scans, a number of groups have been developing “radiomic” approaches for computerized analysis of textural and shape based attributes of the lesion^6–9^. The primary assumption behind these approaches is that there are subtle microarchitectural differences within the nodule, as also shape differences (e.g. malignant nodules are more speculated and benign nodules smooth) that can be captured via corresponding radiomic measurements on chest CT scans. In^10^, Liu *et al*., showed that radiological image traits (quantitative features) were useful in predicting malignancy in lung nodules. The group studied a cohort of 172 patients who had low-dose CT images, with 102 and 70 patients grouped into training and validation cohorts, respectively. A set of 24 radiological traits were systematically scored and a linear classifier was built to identify the possible associations with malignancy. The best feature set including short axis, contour, concavity, and texture yielded an AUC of 0.88 in predicting malignancy in primary nodules in a validation cohort of 70 patients. In^11^ the authors found an association between shape (e.g. surface area, volume and surface to volume ratio), textural and intensity features extracted from CT scans with underlying gene-expression profiles of lung cancer patients. Way *et al*.^12^, showed via leave-one out cross validation approach that a combination of intra-tumoral texture and shape features could distinguish 44 malignant and 52 benign nodules with an AUC of 0.83. In an extension of this study^13^, the same group also incorporated surface features to complement the shape and texture features to improve classification area under the receiver operating characteristic curve (AUC) from 0.82 to 0.85. In another study, McWiliams *et al*., in^14^ attempted to predict the likelihood of malignancy associated with a nodule based off semi quantitative handcrafted nodule features such as the nodule size, spiculation, location and the number of detected nodules along with clinical variables including age, gender, body mass index and family history of lung cancer. This method yielded an AUC of 0.9 for predicting the presence of malignant nodules.

In addition to the previously mentioned approaches, recently deep learning models^15^ and feature embedding tools^16^ have been proposed for automatically learning the most discriminating features from nodules and their immediate surroundings. For example, authors in^17^ presented a deep learning architecture based on the stacked denoising auto-encoder for the differentiation of distinctive types of breast lesions and lung nodules on ultrasound and CT images. They showed that the model outperformed two intensity and texture based CADx methods previously presented in^18^. Setio *et al*.^19^, presented a novel computer aided detection system for pulmonary nodules using multi-view convolutional networks, in which discriminative features were automatically learned from the training data. On 888 scans, their method reached detection sensitivities of 85.4% and 90.1% at 1 and 4 false positives, respectively, per scan. In^20^, the authors proposed a method to find associations between deep convolutional features and multiple human-defined semantic features of CT pulmonary nodules. In^21^, the authors proposed a multi-scale convolutional neural network (CNN) architecture for nodule classification. Their method achieved an 86.84% classification accuracy and outperformed a number of well established textural descriptors. In^22^, deep learning was employed for detecting active lesions exclusively from CT images. The resulting precision and recall (0.79 ± 0.18, 0.57 ± 0.18), was found to be worse compared to using a combined PET/CT exam (0.93 ± 0.13, 0.68 ± 0.27). In^23^, Teramoto *et al*., proposed an improved FP-reduction method for the detection of pulmonary nodules in PET/CT images by means of a CNN. False positives were significantly decreased from 72.8 to 4.9 FPs/case. However, the sensitivity decreased from 97.2% to 90.1%. In the LUNA16^24^ challenge involving pulmonary nodule detection, the leading solutions employed CNNs. Finally, Ciompi *et al*.^25^, presented a deep learning based method for detection of different pulmonary nodules types including solid, calcified, part-solid, non-solid, perifissural and spiculated. Apart from nodule detection, there is a growing interest in the use of deep learning models for diagnosis of lung nodules on CT scans^17^. However, it remains to be seen how the quality of the annotations and the number of training exemplars will ultimately affect the performance of these networks.

It is well established that tumor vasculature is on account of angiogenesis, the process by which tumors grow new blood vessels for nutrient and oxygen supply, and metastasis. While benign tumors are also associated with vasculature, recent findings suggest differences in vascular morphology for benign and malignant tumors^26^. Malignant lesions tend to affect regional changes by modulating to vessel shape and tortuosity. Contortions in the vessel tortuosity appear during the tumor development process and affect initially healthy vessels which spread beyond the confines of the tumor margins. For instance Shelton *et al*.^27^, in a study involving quantitative morphologic analysis of tumor vessels in mice using Acoustic Angiography showed that vascular tortuosity was significantly more pronounced in tumors compared to normal controls. In a pioneering study^28^, Bullitt *et al*., investigated the ability of computer extracted features of vessel morphology to predict the presence of a benign or malignant brain tumor on magnetic resonance angiography (MRA) scans. The same group in^29^ found that the quantitative measurements of vessel morphology from MRA scans could also provide useful insights into brain tumor development and response to therapy. The findings from^28,29^ and^27^ therefore beg the following two questions. Firstly, whether there are significant differences in vessel tortuosity between lung granulomas and adenocarcinomas. Secondly, whether computerized features of vessel tortuosity can be extracted from routine clinical non-contrast CT scans and whether these measurements can enable discrimination of granulomas and adenocarcinomas.

In this study, we present quantitative vessel tortuosity (QVT), a novel CT imaging biomarker to distinguish between benign granulomas and adenocarcinomas on routine non-contrast lung CT scans. QVT is based off the idea that benign lesions like granulomas tend to have a less tortuous vasculature compared to adenocarcinomas.

**Figure 1:**
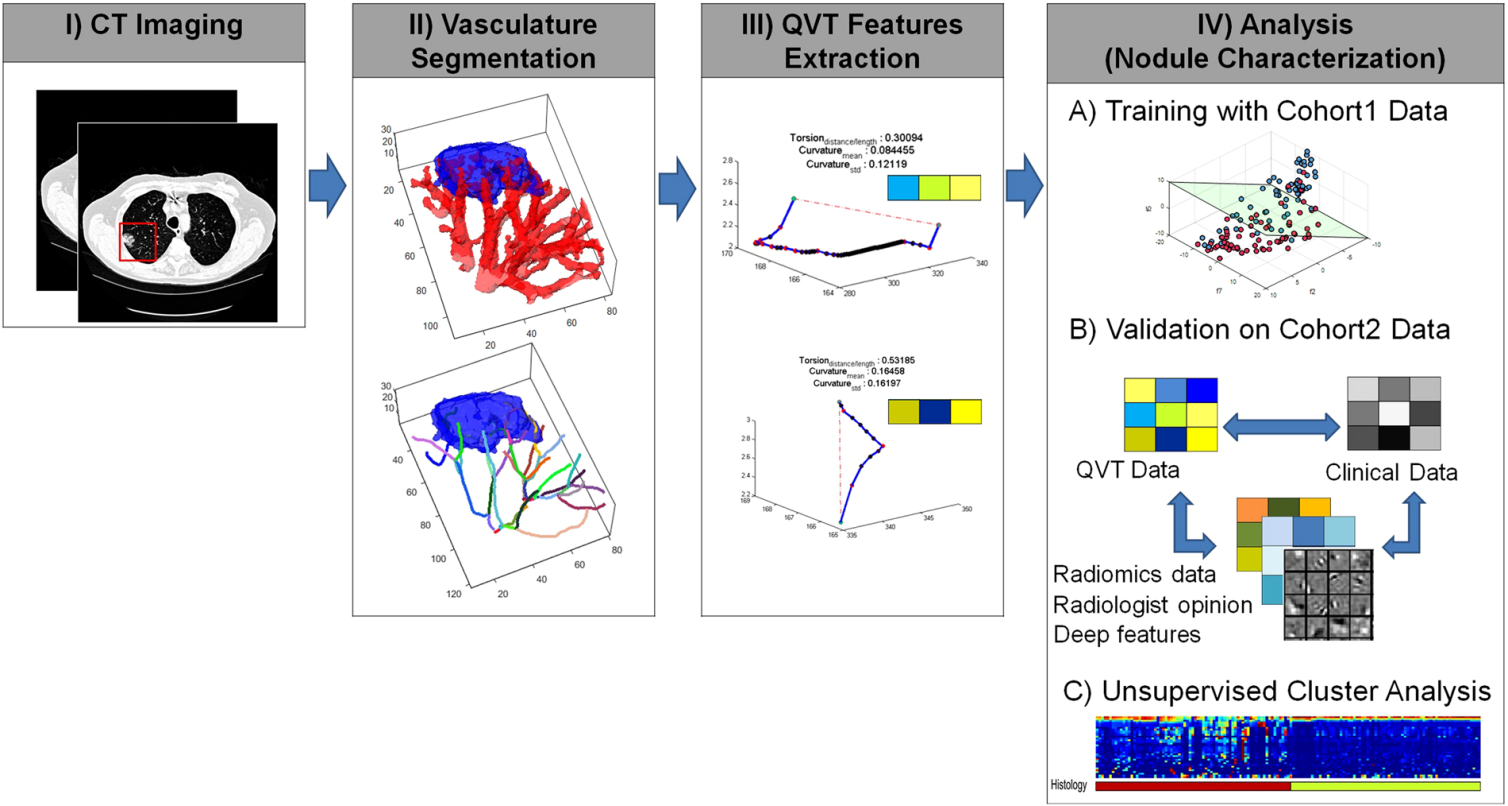
Illustration of workflow for capturing QVT features from chest CT scans. The goal of processing flow is to determine the malignancy probability for the input nodule in a CT image. In step 1, a 3D volume of interest was manually defined around the nodule of interest. In step 2, the associated vasculature was segmented using a 3D region growing algorithm, following which center lines of the vessels are extracted by the fast marching algorithm. In step 3, a set of 35 QVT features were extracted. Step 4 includes data analysis in which the stable and discriminating QVT features were used to train machine learning classifiers to predict the risk of nodule malignancy in the validation set. Unsupervised cluster analysis is then employed to find the association of clinical outcome with the QVT features. These features were also evaluated via human-machine comparison studies and were also compared against the predictive performance of texture and shape radiomics as well as deep features.

Our study comprised of CT scans of 290 patients from two different institutions, one cohort for training (N = 145) and the other (N = 145) for independent validation. All patients had previously undergone surgical wedge resection based off suspicious findings on radiology and hence histopathologically confirmed diagnosis was available for all lesions. A 3D volume of interest was manually defined around the nodule of interest and the associated vasculature was segmented using a 3D region growing segmentation method. A set of 35 QVT features capturing the torsion, curvature and branching statistics of the vessels associated to the nodules were extracted from non-contrast diagnostic CT images. The most discriminating of the 35 QVT tortuosity features were established via feature selection and unsupervised cluster analysis. The features so identified were further pruned based off their stability and reproducibility in the RIDER dataset, a cohort of same day test-retest lung CT scans. The stable and discriminating features thus identified, were used to train machine learning classifiers designated to predict the risk of malignancy to each nodule in the validation set. We perform an exhaustive and rigorous evaluation of our approach with extensive human-machine comparison studies involving two different human readers. Additionally, tortuosity was also quantitatively compared on the validation set against the performance of the state of the art texture and shape features as well as deep features which were automatically learned from nodule regions. The sensitivity of the approach was evaluated as a function of slice thicknesses of CT scans that varied from 1 to 5 mm. The workflow of the current study is presented in Fig. 1. As may be appreciated from Fig. 1, in step 1, a 3D volume of interest was manually defined around the nodule of interest. In step 2, the associated vasculature was segmented using a 3D region growing algorithm, following which center lines of the vessels are extracted by a fast marching algorithm. In step 3, a set of 35 QVT features were extracted. Step 4 includes data analysis in which the stable and discriminating QVT features were used to train machine learning classifiers to predict the risk of nodule malignancy in the validation set. Unsupervised cluster analysis was then employed to find the association of clinical outcome with the QVT features. These features were also evaluated via human-machine comparison studies and were also compared against the predictive performance of texture and shape radiomics as well as deep features.

## Results

### Identifying the most discriminating QVT features and cluster analysis

The top 12 most predictive QVT features were identified using the minimum redundancy-maximum relevance (mRMR) feature selection algorithm^30^. A comparison of feature selection strategies including mRMR, least absolute shrinkage and selection operator (LASSO) and principal component analysis (PCA) is provided in Table S5 of the Supplemental Material section. Since the nodule vasculature is comprised of several vessel branches, where each branch is in turn comprised of several points in a 3D space, these QVT features capture first order statistics associated with the average curvature values of the branches. Other QVT features include first order statistics of the maximum curvature values of the branches, volume of the vasculature and the ratio of vasculature volume to the 3D volume of the interest. Additional features include the histogram of curvature and torsion values associated with 3D points on the vessel branches. A detailed description of the features can be found on Table S1 in the Supplementary File.

**Figure 2:**
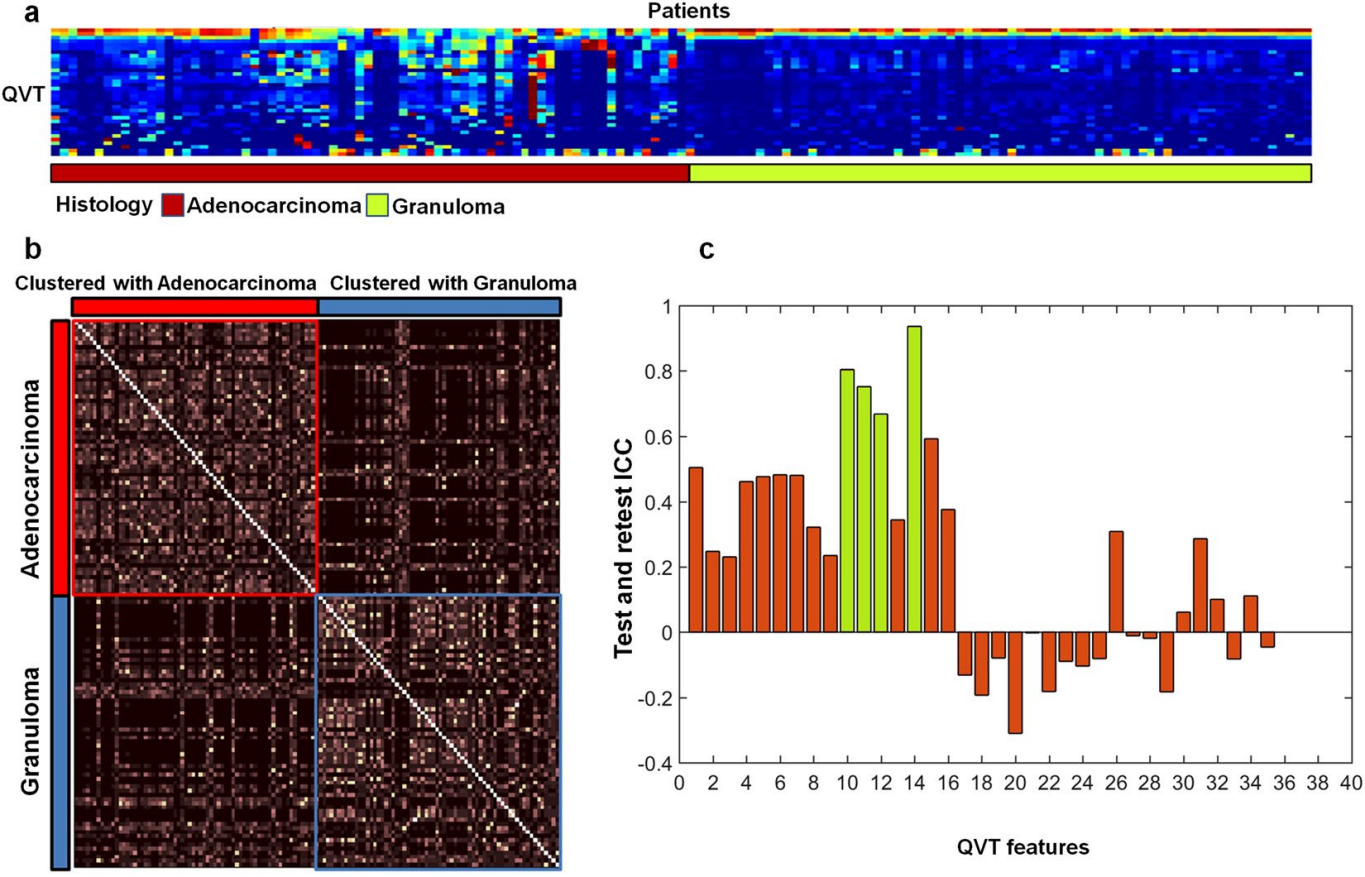
(**a**) QVT heatmap showing differential expression for the adenocarcinomas and granulomas within *D*_1_. X axis (columns) corresponds to the patients and Y axis (rows) corresponds to the most discriminating QVT features. Colors represent feature values normalized between [0, 1]. A number of identified QVT features showed significant differential expression between the adenocarcinomas and granulomas for the patients in *D*_1_. (**b**) Two dominant clusters emerge by applying ensemble clustering of the QVT features for the patients in *D*_1_. Performing iterative clustering with different parameters and counting the co-occurrences of the patient pairs falling into a same cluster resulted in roughly 75% of the cases within each cluster being assigned to a single category. Pixel intensities correspond to the co-occurrence of a pair of patients clustered within the same group. (**c**) The ICC values for all 35 QVT features as assessed on the RIDER dataset. ICC > 0.7 corresponds to highly reproducible features. ICC > 0.4 suggests moderate stability.

Figure 2(a) illustrates a feature expression heat map of the most discriminating QVT features for the granulomas and adenocarcinomas in *D*_1_. The QVT feature expression is illustrated on the Y-axis and the X-axis corresponds to the different patients in training set (*D*_1_). As may be observed from Fig. 2(a), a number of the QVT features showed statistically significant differential expression between the adenocarcinomas and granulomas for the patients in *D*_1_. The p-values computed under the null hypothesis that there was no significant difference between the 12 QVT features between adenocarcinomas and granulomas were found to be <0.05. The actual p-values for this comparison for the 12 QVT features for the studies in *D*_1_ are listed in Table S1 of the Supplemental Material section.

Unsupervised clustering of the patients in *D*_1_ was carried out to find a potential association between the predicted number of clusters and the clinical data (i.e, the histopathologic diagnosis of the lung nodule). A *χ*^2^ test performed on clustering results revealed a significant association between patients cluster labels and the histopathologic diagnosis of these patients in *D*_1_ with p-value = 3.5*e*^−7^. Additionally, unsupervised ensemble clustering^31^ of the patients described with the top 12 QVT features, yielded two dominant patient clusters. As may be appreciated from Fig. 2(b), evaluation of the two dominant clusters identified via unsupervised ensemble clustering revealed that roughly 75% of the cases within each cluster correspond to a single category (i.e. adenocarcinomas or granulomas).

**Figure 3:**
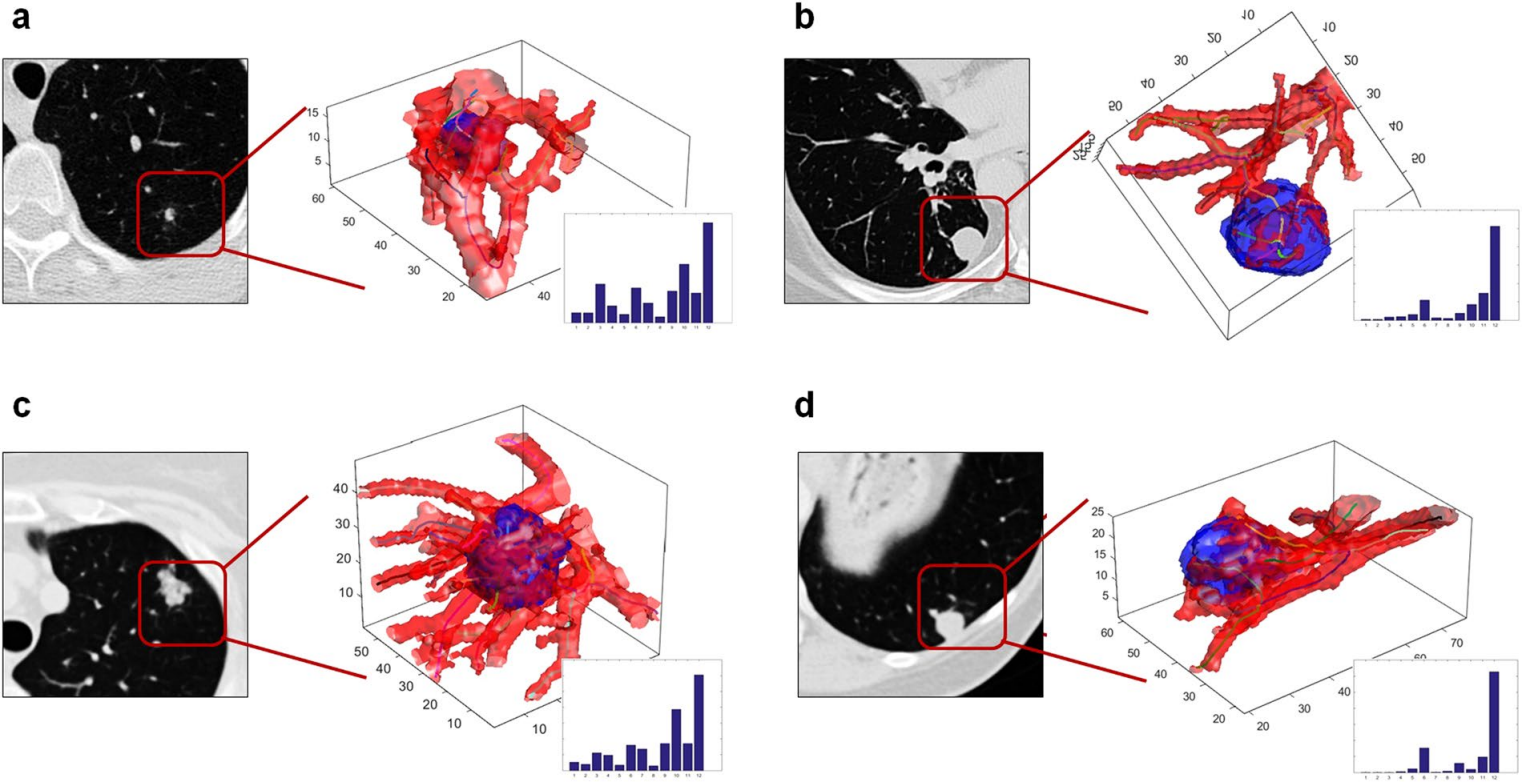
3D rendering of 2 adenocarcinomas (**a**,**c**) and 2 granulomas (**b**,**d**). Corresponding feature vector of each nodule in the form of a bar graph is illustrated in the bottom left of each individual panel. The corresponding height of each column is a reflection of the expression value for each of the top 12 discriminating and QVT features.

Figure 3 illustrates CT scans corresponding to 2 adenocarcinomas and 2 granulomas. Additionally the figure also illustrates the 3D renderings of nodule vasculature (in red) for the two cases. The corresponding QVT feature vector for each nodule is illustrated in the form of a bar graph in the bottom left of each individual panel. The columns of the bar graph represent the most informative QVT features and the height reflects the corresponding quantitative feature expression. As may be appreciated in Fig. 3, the first 4 QVT features appear to significantly over-express in the case of the adenocarcinomas (Fig. 3(a,c)) and appear to mostly under-express for the granulomas (Fig. 3(b,d)).

### Stability analysis of the QVT features identified as most discriminating

Among the 12 QVT features identified via feature selection, first order statistics pertaining to the maximum curvature value of the branches and the ratio of the vasculature volume to the 3D volume of interest were identified as being most stable. These features had an average Intraclass Correlation Coefficient (ICC) = 0.83 ± 0.09, suggesting a high degree of reproducibility for the same day test-retest cases within the RIDER datset. The ICC values obtained for all 35 QVT features on the RIDER dataset are illustrated in Fig. 2(c).

### Evaluating the ability of the QVT features to distinguish adenocarcinomas from granulomas

**Figure 4:**
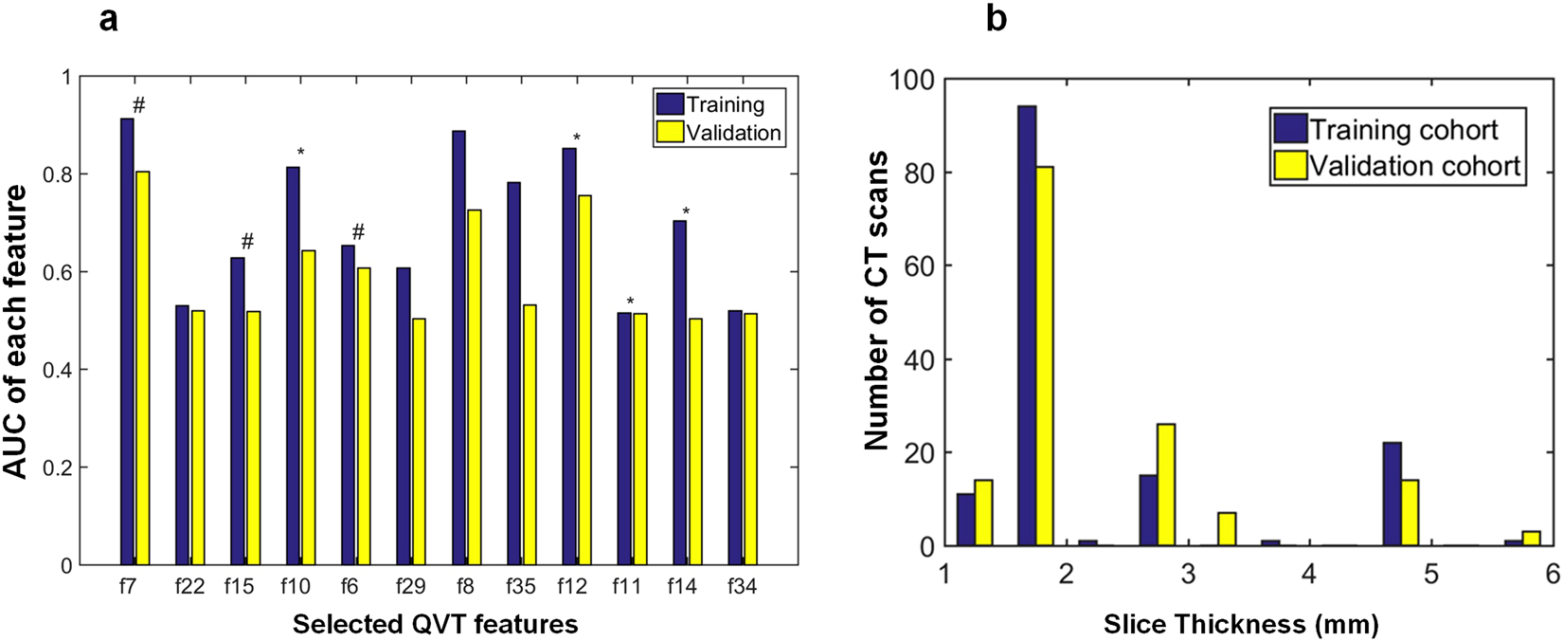
(**a**) AUC values for each individual informative and stable feature in the training (*D*_1_) and validation (*D*_2_) sets. Features with high and moderate stability are identified with a * and # signs respectively. (**b**) The distribution of slice thicknesses on both *D*_1_ (blue) and *D*_2_ (yellow) respectively. X and Y axes respectively represent the slice thickness in millimeters and the number of CT scans with corresponding slice thickness in both *D*_1_ and *D*_2_, respectively.

**Figure 5:**
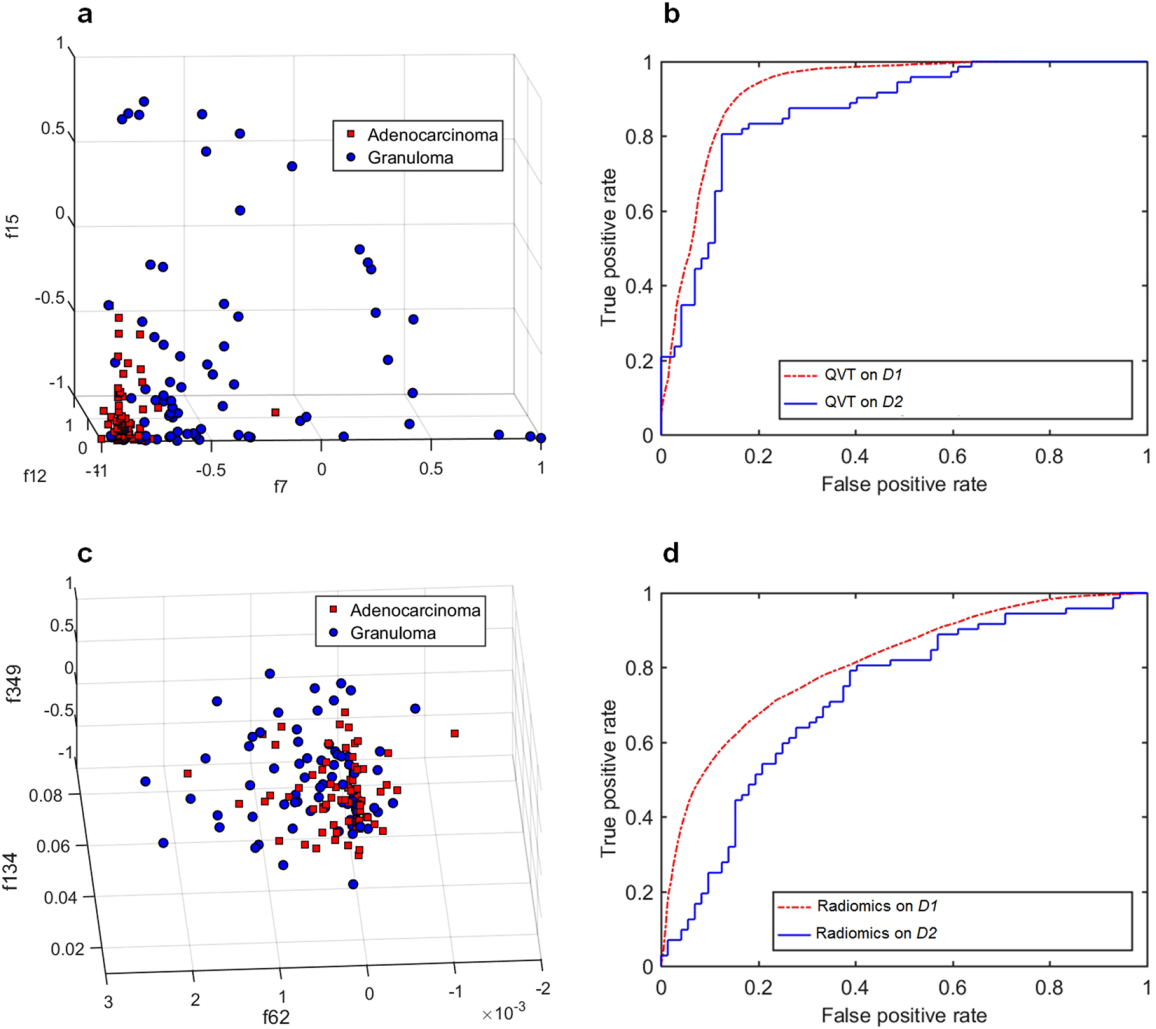
(**a**,**c**) The 3D cluster plot of adenocarcinomas (red dots) and granulomas (blue dots) within the training set (*D*_1_) in a reduced three dimensional space of the QVT features (top row) and well-known nodules shape and texture radiomic features (bottom row). (**b**) ROC curves of *C*_*QVT*_ trained on *D*_1_ and validated on *D*_2_ based off QVT features. (**d**) ROC curves of *C*_*rad*_ trained on *D*_1_ and validated on *D*_2_ based off shape and texture radiomic features respectively. *C*_*QVT*_ and *C*_*rad*_, respectively, yielded an AUC of 0.85 and 0.73 for cases in *D*_2_.

The tortuosity based classifier (*C*_*QVT*_) yielded an AUC of 0.85 for the cases in validation set (*D*_2_). The corresponding AUC for *D*_1_ was 0.94 ± 0.02. Figure 4(a) shows the AUC values for each individual feature from among the top 12 selected features for both *D*_1_ and *D*_2_. Figure 5(b) illustrates the ROC curves for *C*_*QVT*_ on both *D*_1_ (red) and *D*_2_ (blue) for the support vector machine (SVM) classifier trained with QVT features. The SVM classifier outperformed the k-nearest neighbors (KNN) and Naive Bayes classifiers. Figure 5(a) represents the 3D cluster plot of adenocarcinomas (red dots) and granulomas (blue dots) within *D*_1_ in a 3D space involving the three most discriminating and stable QVT features identified on *D*_1_. As may be appreciated in Fig. 5(a), two classes of nodules within *D*_1_ appear to be clearly separable.

The impact of slice thickness on the performance of the *C*_*QVT*_ classifier was also evaluated. The distribution of the cases with differing slice thickness in both *D*_1_ and *D*_2_ is shown in Fig. 4(b). Table 1 illustrates the performance of *C*_*QVT*_ and *C*_*rad*_ classifiers in terms of AUC as a function of the slice thickness parameter on *D*_2_. *C*_*rad*_ corresponds to a classifier trained with established radiomic features pertaining to nodule texture and shape on *D*_1_. From Table 1, it can be seen that, the AUC values for the QVT features tends to drop slightly with increasing slice thickness.

**Table 1:** A breakdown of the AUC performance of the *C*_*QVT*_ and *C*_*rad*_ classifiers as a function of CT slice thickness for the cases in *D*_2_.

Slice thickness criteria	Studies on *D*_2_	*C*_*QVT*_	*C*_*rad*_
*slc* ≤ *mm*	10	0.89	0.67
1 < *slc* ≤ 2	94	0.84	0.73
2 < *slc* ≤ 3	17	0.83	0.75
3 < *slc* ≤ 6	24	0.76	0.79

### Comparing QVT with state of the art radiomic features

*C*_*QVT*_ outperformed the classifier trained with well-known radiomic features (*C*_*rad*_) on *D*_2_. *C*_*rad*_ yielded an AUC of 0.82 ± 0.05 and 0.73 on *D*_1_ and *D*_2_ respectively. Figure 5(d) illustrates the ROC curve of the *C*_*QVT*_ classifier on *D*_1_ and *D*_2_ respectively. Furthermore, in Fig. 5(a,b), it can be seen that there is an improved separation between nodules in the reduced space of QVT features, compared to the nodule derived texture and shape based radiomic features.

### Comparison with deep learning networks

Using the same training and testing sets as in the previous experiments, with LeNet (*C*_*LeNet*_), we achieved a training accuracy of 0.99. The weights after 100 epochs were locked down and used on the test set. The testing AUC was computed to be 0.76. At the operating point, the corresponding accuracy was computed to be 0.737. The performance metric across the training runs are shown in section I of the Supplemental Material. Additionally, the training set accuracy for VGG-19 CNN, trained on the ImageNet database, was 0.94, while the test set accuracy was 0.62. Table 2 shows the AUC values of *C*_*QVT*_, *C*_*rad*_ and deep learning classifier (*C*_*LeNet*_ and *C*_*VGGNet*_) in both the training and validation sets.

**Table 2:** AUC values for *C*_*QVT*_, *C*_*rad*_ as well as deep learning classifiers (*C*_*LeNet*_ and *C*_*VGGNet*_) obtained from both the training *D*_1_ and validation *D*_2_ cohorts.

Classifiers	Mean AUC on *D*_1_	AUC on *D*_2_
*C*_*QVT*_	0.94	0.85
*C*_*rad*_	0.82	0.73
*C*_*LeNet*_	0.99	0.76
*C*_*VGGNet*_	0.94	0.62

### Comparison with human experts

While *C*_*QVT*_ yielded an AUC of 0.85 on *D*_2_, on the same cohort the AUC for the two human readers was 0.61 and 0.60. These results are summarized in Table S4 of the Supplementary File.

### Statistical analysis between patient and CT specific parameters with histopathologic diagnosis of the nodule

Statistical significance test results between patient parameters and clinical outcome for both the *D*_1_ and *D*_2_ cohorts is shown in Table 3. Similarly, Table 4 illustrates the results of significance testing between CT parameters and histopathologic diagnosis of the nodule. The presence of a statistically significant difference was indicated by p < 0.01. ‘Smoking status’ and ‘Age’ were the only patient parameters that were found to be significantly different between adenocarninoma and granulomas in *D*_1_. While in *D*_2_ ‘Age’ was found to be significantly different, no significant differences were identified between ‘Slice Thickness’, ‘Nodule Size’ and ‘Voxel Size’ between the adenocarninomas and granulomas in *D*_1_ or *D*_2_. The effect of scanner variability and voxel size as well as smoking history on the QVT features can be found on section H of the Supplemental Material.

**Table 3:** Statistical significance testing between patients parameters and disease outcome for both training and validation cohorts. The p-values were computed using Students t test for continuous variable and Fishers exact test for categorical data. Statistically significant difference was indicated by p < 0.01.

Characteristic	Training set				Validation set			
	Adeno	Granu	Total	p-value	Adeno	Granu	Total	p-value
**Gender**				**0.31**				**0.50**
Male	27	33	60		31	35	66	
Female	46	39	85		42	37	79	
**Smoking status**				<**0.01**				**0.05**
Yes	53	17	70		43	25	68	
No	2	20	22		8	13	21	
Not Known	18	35	53		22	34	56	
**Ethnicity**				**0.82**				**0.68**
White	41	38	79		43	51	94	
Black	12	12	24		13	19	32	
Other	20	22	42		17	2	19	
**Age (years)**				<**0.01**				<**0.01**
Mean	73.87	62.85	68.36		72.08	61.31	66.7	
Std Dev	10.34	14.2	12.27		10.7	12.54	11.62

**Table 4:** Statistical significance testing between CT parameters and disease outcome for both training and validation cohorts. The p-values were computed using two tailed and unpaired Students t test. Statistically significant difference was indicated by p < 0.01.

Characteristic	Training set				Validation set			
	Adeno	Granu	Total	p-value	Adeno	Granu	Total	p-value
**Nodule Size**				**0.42**				**0.011**
Mean	20.23	19.11	19.67		21.12	17.18	19.15	
Std Dev	7.99	8.96	8.47		10.77	7.18	8.97	
**Slice Thickness**				**0.44**				**0.05**
Mean	2.45	2.6	2.52		2.73	2.35	2.5	
Std Dev	1.14	1.17	1.15		1.4	0.93	1.16	
Median	2	2	2		2	2	2	
**Voxel Size**				**0.65**				**0.05**
Mean	0.73	0.74	0.74		0.70	0.74	0.72	
Std Dev	0.11	0.11	0.11		0.11	0.12	0.12	
Median	0.72	0.73	0.72		0.68	0.72	0.70	

### Comparing nodule adjacent QVT with normal lung QVT

**Figure 6:**
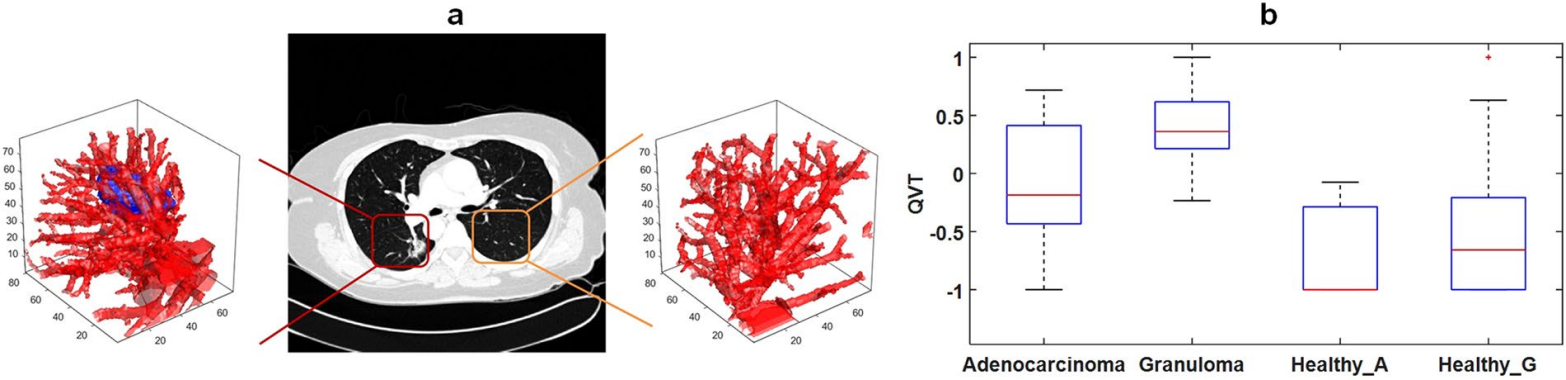
(**a**) 3D renderings of a non-healthy region (left panel including a nodule) and corresponding healthy region (right panel without a nodule). (**b**) The top ranked QVT feature values of the non-healthy regions including adenocarcinomas and granulomas and the healthy regions including Healthy_A and Healthy_G. Healthy_A corresponds to the regions in the opposite lung of adenocarcinomas which had no nodules on them while Healthy_G corresponds to the regions in the opposite lung of granulomas having no nodules on them.

The QVT differences between the healthy and non-healthy regions were compared. The nodule-adjacent QVT values were significantly different (p < 0.05) between the adenocarcinomas and the granulomas. Interestingly, there was no significant difference between the QVT values of the healthy regions taken from patients with adenocarcinoma (Healthy_A) and healthy regions taken from patients with granulomas (Healthy_G). Also QVT was found to be significantly different between the healthy and non-healthy regions. The p-value of the t-test significance analysis between adenecarcinomas and Healthy_A was 0.017 while it was 0.0016 between granulomas and Healthy_G. Figure 6(a) illustrates a nodule (center panel), a 3D rendering of nodule associated vasculature (left panel) as well as 3D rendering of vasculature in a corresponding healthy region (right panel). Figure 6(b) shows the top ranked QVT feature values of the non-healthy regions including adenocarcinomas and granulomas and the healthy regions, Healthy_A and Healthy_G.

### Association of the nodule location and diagnostic class

**Figure 7:**
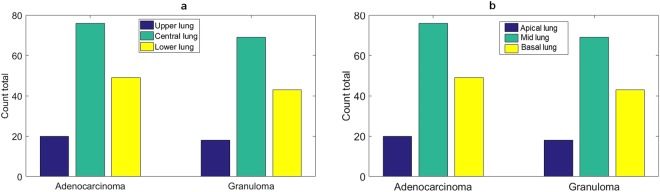
The distribution of the nodules based on (**a**) their position in 2D transverse lung plane which include upper, central and lower lung nodules. (**b**) Their position along z-axis including apical, mid or basal lung regions.

A frequency plot for the nodule position for both adenocarcinomas and granulomas is shown in Fig. 7. In the 2D transverse plane we identified whether a nodule was located within central, upper or lower lung regions (Fig. 7a). Additionally, in the sagittal plane (z-axis), we determined whether a nodule was located in the apical, mid or basal lung regions (Fig. 7b). Performing *χ*^2^ test^32^ between nodule position and diagnostic class revealed that there was no significant association between the nodule position (in both 2D and along z-axis) and its diagnostic class. The corresponding p-values are shown in the Table 5. Additional details can be found in Section F.1 of the Supplementary Material.

**Table 5:** The p-values corresponding to the *χ*^2^ test between the diagnostic class of a nodule and its relative position in 2D and 3D (along z-axis). p-value < 0.01 was considered as corresponding to a significant association between the spatial location of a nodule and its diagnostic class.

	2D transverse lung position vs Nodule class		Position along z-axis vs nodule class	
	Training set	Validation set	Training set	Validation set
**p-value**	*left*: 0.05 *right*: 0.92	*left*: 0.49 *right*: 0.64	*left*: 0.06 *right*: 0.38	*left*: 0.75 *right*: 0.015

### Impact of spatial location of a nodule on its corresponding QVT feature measurements

As described in Section F.2 of the Supplementary Material, a set of control regions were defined to determine whether a nodule was located in the a) apical or lower lung portions, b) Gravity dependent or non-dependent portions of the lung and c) peripheral or central regions. Applying, statistical significance testing to the QVT measurements of the nodules belonging to the aforementioned regions revealed that QVT features were not significantly different between apical and lower lung regions. QVT features were however found to be significantly different for nodules located in the dependent and non-dependent lung regions. The statistically significantly different features included the mean torsion of the vessel branches for adenocarcinomas, and the ratio of vasculature volume to its bounding box for granulomas. Finally, 5 QVT features (branching count of the vasculature, 1 feature of curvature histogram, 3 features of torsion histogram) were significantly different between adenocarcinomas of central and peripheral regions. For the granulomas within the same regions, 3 QVT features (mean and standard deviation values of the torsion of the vessel branches, 1 feature of torsion histogram) were found to be significantly different. A more detailed description of these findings is in Section F.2 of the Supplementary Material.

### The impact of smoking status on QVT measurements of healthy lung

**Figure 8:**
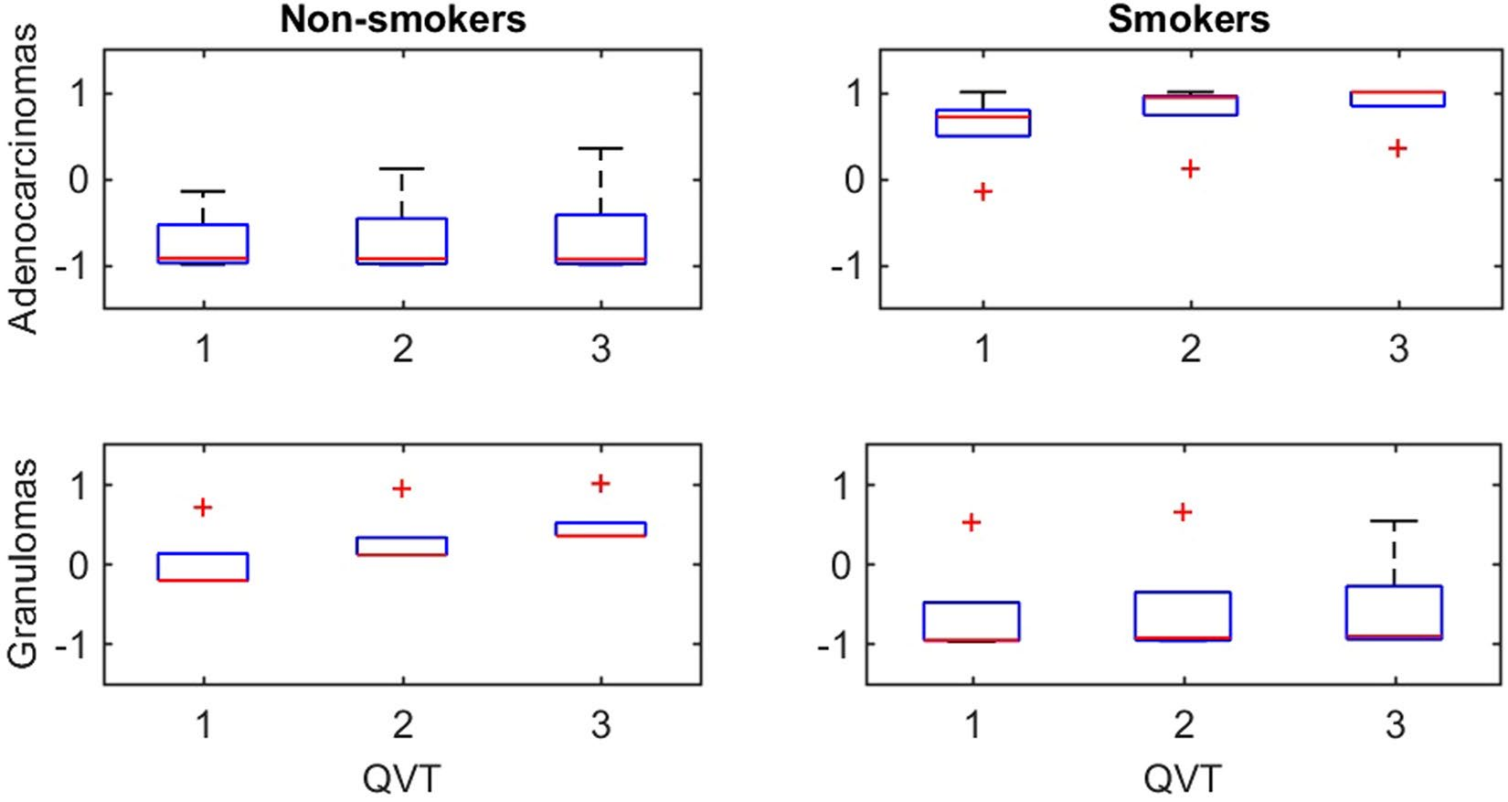
A set of 20 cases including 10 adenocarcinomas and 10 granulomas was selected. Within each group, five were non-smokers and five were smokers and had at least 50 pack years. Three QVT features were statistically different (p < 0.01) between smokers and non-smokers, both within the set of adenocarcinomas and granulomas.

In this section, we studied the effect of smoking status on the QVT measurements on healthy lung regions. A preliminary analysis was conducted. We selected 20 cases including 10 adenocarcinomas and 10 granulomas. Within each group, five were non-smokers and five were smokers and had at least 50 pack years. For each case we extracted the QVT features from healthy regions according to the method described in the Section entitled “Comparing nodule adjacent QVT with normal lung QVT”. Three QVT features were found to be significantly different between smokers and non-smokers, both within patients with adenocarcinomas and granulomas. These features (all of which had a p < 0.01) included mean, std and max values of the averaged curvature of the vasculature branches. The first row of Fig. 8 illustrates that the QVT features of adenocarcinomas were different between smokers and non-smokers. Similarly, the second row shows the difference between smokers and non-smokers with granulomas.

## Discussion

The five-year survival rate following diagnosis of lung cancer in the U.S. is roughly 16%^33^. In contrast, the survival rate is above 50% when the disease is diagnosed when it is still localized^34^. Unfortunately, only 15% of lung cancers are diagnosed at an early stage^35^. While substantial evidence exists that CT enables early detection of lung cancers and hence can be effective in decreasing the mortality associated with the disease. Radiologist interpretation of lung CT scans is subject to inter-reader variability^36^. Additionally radiologist interpretations tend to result in a large number of lung nodules as being identified as either indeterminate or suspicious. For example, in a recent study^37^, investigators from eight Veteran centers across the U.S. screened more than 2000 Veterans over two years using criteria from the national lung screening trial (NLST). Among the 2106 Veterans screened, a total of 1257 (59.7%) had nodules, of which 1184 (56.2%) required tracking. Nearly all of the positive results were negative for cancer, producing a false-positive rate of 97.5% from radiologist based CT interpretations of the CT exams. Consequently, there has been substantial recent interest in the application of radiomic based approaches for non-invasive characterization of lung nodules on CT scans. However most of these approaches have relied on textural^38,39^ and intensity or shape^40^ based attributes which may not necessarily provide sufficient discriminability between tumor confounding benign pathologies such as granulomas.

Recent findings in pre-clinical and clinical studies suggest that tumor vasculature might have different morphologies in benign versus malignant conditions^27^. Some studies^28,29^ have suggested that there are significant differences in tortuosity and convolutedness of tumor vasculature between benign and malignant lesions. Motivated by these findings, in this study we presented QVT, a novel CT imaging biomarker to distinguish between benign granulomas and adenocarcinomas on routine non-contrast lung CT scans. QVT represents a new set of mathematically derived measurements that are based off the idea that benign lesions like granulomas tend to have a less tortuous vasculature compared to adenocarcinomas. To the best of our knowledge, this is the first attempt to quantitatively capture and evaluate the role of vessel tortuosity to discriminate between granulomas and adenocarcinomas on lung CT images. The robustness of the QVT was examined via the independent validation and also ensuring that the training and validation cohorts comprised of cases from two different sites. We also performed an exhaustive and rigorous evaluation of our approach with extensive human-machine comparison studies involving two different human readers. It was demonstrated that, QVT performed (AUC = 0.85) substantially better compared to two human expert readers (AUC = 0.61, 0.60). Additionally, our experiments also revealed that QVT features yielded a better classification AUC performance compared to well-known nodule shape and texture radiomic features as well as deep features learned with a convolutional neural network. A recent study by He *et al*.^41^, have suggested that radiomic features tend to be affected by the choice of scanner, reconstruction kernel, and slice thickness. Differences in scanner and CT parameters may affect the reproducibility of radiomic features. In this work, we chose to explicitly evaluate the stability of QVT features using the RIDER test set and found that from among the initial set of 35 QVT features, 4 were highly stable (ICC > 0.7) while 6 were found to have moderate stability (ICC > 0.4). Highly stable features included first order statistics of the maximum curvature values of the branches and the ratio of vasculature volume to the 3D volume of the interest. The moderately stable features corresponded to the first order statistics of the torsion values of the branches. The majority of the features corresponding to the histogram of curvature and torsion measurements were found to not be stable. The segmentation quality of the vasculature could also be another potential source of feature instability. Future work will be necessary to study the effect of segmentation errors between repeated scans on feature instability. The sensitivity of the approach was also evaluated in terms of slice thickness of the CT scans. The AUC values for the QVT features tended to drop slightly with increasing slice thickness. This may be due to the loss of spatial resolution with a corresponding increase in slice thicknesses^41^. Even though the performance tapered off towards the higher slice thickness, *C*_*QVT*_ yielded better AUC compared to shape and texture radiomic features.

A number of studies have looked at the problem of distinguishing benign from malignant lesions on CT scans^10,13,42,43^, but only a few of these studies were aware of have explicitly looked at the problem of distinguishing granulomas from adenocarcinomas^40,44^. For instance, Shah *et al*.^45^, achieved AUC values between 0.68 and 0.92 with 48 malignant and 33 benign nodules, but the benign nodules did not exclusively comprise granulomas, arguably the most challenging benign tumor confounder on CT and PET scans. Dennie *et al*.^46^, employed Haralick-related texture features on 55 nodules to discriminate granulomas from primary lung cancer (including adenocarcinoma and squamous cell cancer). Although the approach reported an AUC = 90.2%, it was not validated on an independent test set. The role of shape radiomic features in distinguishing adenocarcinomas and granulomas was previously studied by us in^40^. On a cohort comprising 149 cases, the shape based classifier yielded an AUC of 0.72.

One common attribute associated with the majority of previous radiomic related approaches for lung nodule characterization is the fact that they involve features pertaining to the nodule region alone and not associate nodular structures. However, a growing body of evidence suggests that features pertaining to peri-nodular regions and tumor adjoining areas may be critical in characterizing disease presence^47^, aggressiveness^48^ and treatment response^49^. Braman *et al*., in^49^ showed that radiomic features of the peri-tumoral region in breast magnetic resonance imaging (MRI) scans were predictive of pathologic complete response (pCR) in breast cancers, the peri-tumoral radiomic features being more predictive of pCR compared to the intra-tumoral features. Prasanna *et al*.^48^, showed that texture features of the peri-tumoral habitat in brain tumors on MRI was prognostic of long versus short term survival. However, these approaches were primarily focused on radiomic based approaches pertaining to texture and heterogeneity characterization within the tumor habitat. Our novel approach goes beyond characterization of texture patterns of the tumor habitat, focusing instead on the morphology of the nodule vasculature. While previous studies have noted differences in vasculature morphology between benign and malignant presentations^27,28^, QVT represents a radiomic based formulation to quantitatively capture and describe vessel tortuosity. The demonstrated QVT differences among normal vs abnormal lung as well as among adenocarcinomas and granulomas suggest that QVT could potentially be considered as an imaging biomarker. While there is certainly promise for QVT to be considered as a potentially predictive imaging marker to distinguish between granulomas and adenocarcinomas, the bar for validating it as a biomarker is higher. In other words, additional criteria^50–52^ need to be met for QVT to be established and validated as a biomarker.

Our study did have its limitations. As mentioned earlier, the segmentation quality of the vasculature could be a potential source of feature instability. Further work is needed to study the effect of segmentation errors on possible instability of the QVT features. Additional validation of the method with larger datasets from multiple external sites is another future direction. Further, our approach was focused on granulomas and adenocarcinomas alone. Even though granulomas represent the most common confounder of adenocarcinomas, to showcase the utility of QVT as an imaging biomarker that is relevant for the lung cancer screening population, we would need to evaluate its ability to distinguish between other benign and malignant presentations such as lymphoma, fibroma and hamartoma. Another limitation of this study is that we focused solely on a single reader identified nodule for each study, i.e. the nodule that had been excised or biopsied. An obvious extension would be to analyze the vasculature for multiple nodules in the case of presentations with multi-nodular disease. Furthermore, additional analyses of QVT differences in normal and abnormal lung as well as the evaluation of the impact of nodule position and smoking history on QVT have been performed on small sub-sets. Therefore, as a future direction, an extensive validation and confirmation of the initial findings might need to be performed on additional datasets. In spite of the aforementioned limitations, our initial findings suggest that QVT could potentially serve as a non-invasive imaging biomarker to distinguish granulomas from adenocarcinomas on non-contrast CT scans.

## Methods

### Dataset

In this retrospective study, we collected patients from two sites. Inclusion criteria for these cases were the existence of a diagnostic or a screening CT exam and the presence of a radiographically confounding nodule, one that had previously triggered a surgical intervention, either in the form of a bronchoscopic or CT guided biopsy or a surgical wedge resection. Additionally the inclusion criteria included the histopathologic confirmation of whether the nodule was malignant or benign. Only granulomas and adenocarcinomas were included in this study. All scans acquired for this study were collected as part of an Institutional Review Board-approved, health insurance portability and accountability act of 1996 (HIPAA)-compliant protocol. The inclusion and exclusion criteria flowchart is shown in Fig. S3 of the Supplementary Material. All scans were in digital imaging and communications in medicine (DICOM) format and de-identified to remove patient header information. Need for an informed consent was waived. Histology was confirmed by an thoracic pathologist based off visual interrogation of the biopsied or surgically resected specimen. A total of 290 patients were included in this study. A set of 125 cases including 64 adenocarcinomas and 61 granulomas were acquired from site 1. Whereas, the cases from site 2 comprised of 165 cases including 81 adenocarcinomas and 84 granulomas. All cases were bundled together and split into training (*D*_1_ = 145) and validation (*D*_2_ = 145) cohorts in a blinded way with the only caveat that the training set have a 50-50 split of adenocarcinomas and granulomas. Details of the datasets are provided in Table 6. CT scans were acquired on both Philips and Siemens scanners. The scans were acquired with a tube current between 96 and 426 mAs and voltage of either 100 or 120 kVp.

**Table 6:** Details of the distribution of the granulomas and adenocarcinomas within *D*_1_ and *D*_2_.

Data set	Adenocarcinomas	Granulomas	Total
*D*_1_	73	72	145
*D*_2_	73	72	145
Total	146	144	290

### Nodule detection and Segmentation of Vasculature

The nodule of interest was manually identified on the CT scan by an expert cardiothoracic radiologist with 20 years of experience. A region of interest (ROI) was manually placed on the nodule of interest by the same radiologist across all contiguous slices on which the nodule was visible via a hand annotation tool in 3D-Slicer software^53^. Each scan included one nodule of interest. The impact of nodule’s location on the predictions via the QVT classifier were also determined. This involved identifying whether there was an association between nodule position and its diagnostic class. Additional details on these correlative studies are presented in section F of the Supplemental Material.

#### Segmentation of vasculature associated with a nodule of interest

**Figure 9:**
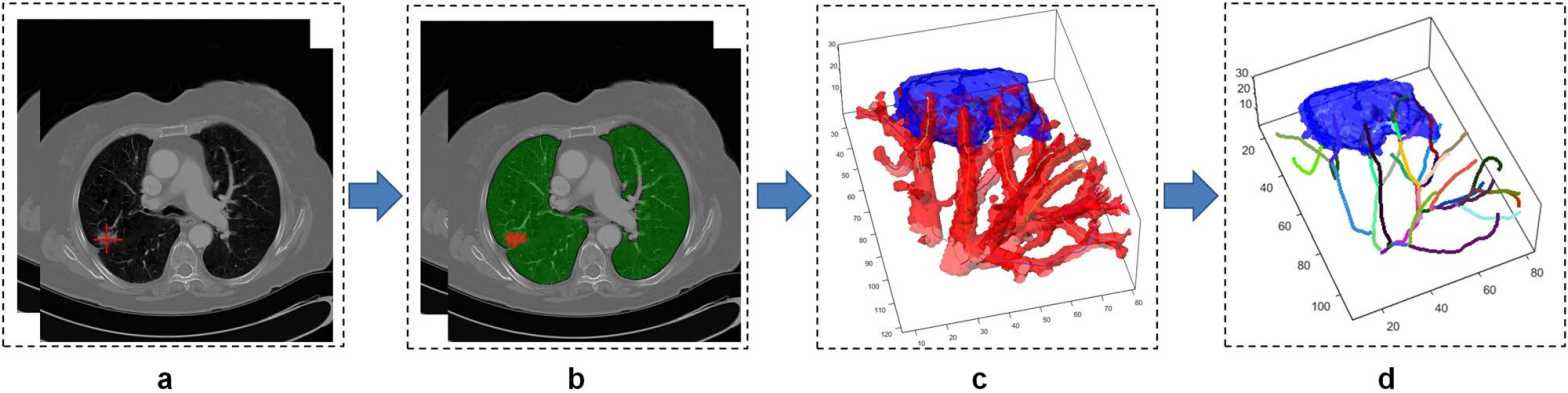
(**a**) A nodule of interest is identified by the radiologist. (**b**) Lung regions are automatically isolated from surrounding anatomy using a multi-threshold based algorithm^54,55^. (**c**) The vasculature is segmented in a 3D volume via a region growing algorithm^57^. (**d**) The center lines and branches of the vasculature are detected by a fast marching algorithm^58^. The fast marching algorithm enables determination of the skeleton of the nodule vasculature using the fast-marching distance transform^71^. The QVT features are then extracted from the center lines of the vessels.

In the first step, lung regions are isolated from the surrounding anatomy using a multi-threshold based algorithm previously presented in^54,55^. In the second step, a region growing algorithm is employed for the segmentation of the nodule vasculature^56^. The center of gravity of the segmented nodules is used as the initial seed points for the region growing algorithm^57^. Within the nodule volume, seed points were initialized at random locations. Based off the intensity similarity of the seed points and surrounding pixels, an initial region iteratively grows to incorporate nodule and vasculature related voxels. A fast marching algorithm^58^ is then employed to identify the center lines of the 3D segmented vasculature^59^. Figure 9 illustrates the process of nodule detection and segmentation of vasculature.

#### Sensitivity of QVT features to minor vasculature segmentation errors

**Figure 10:**
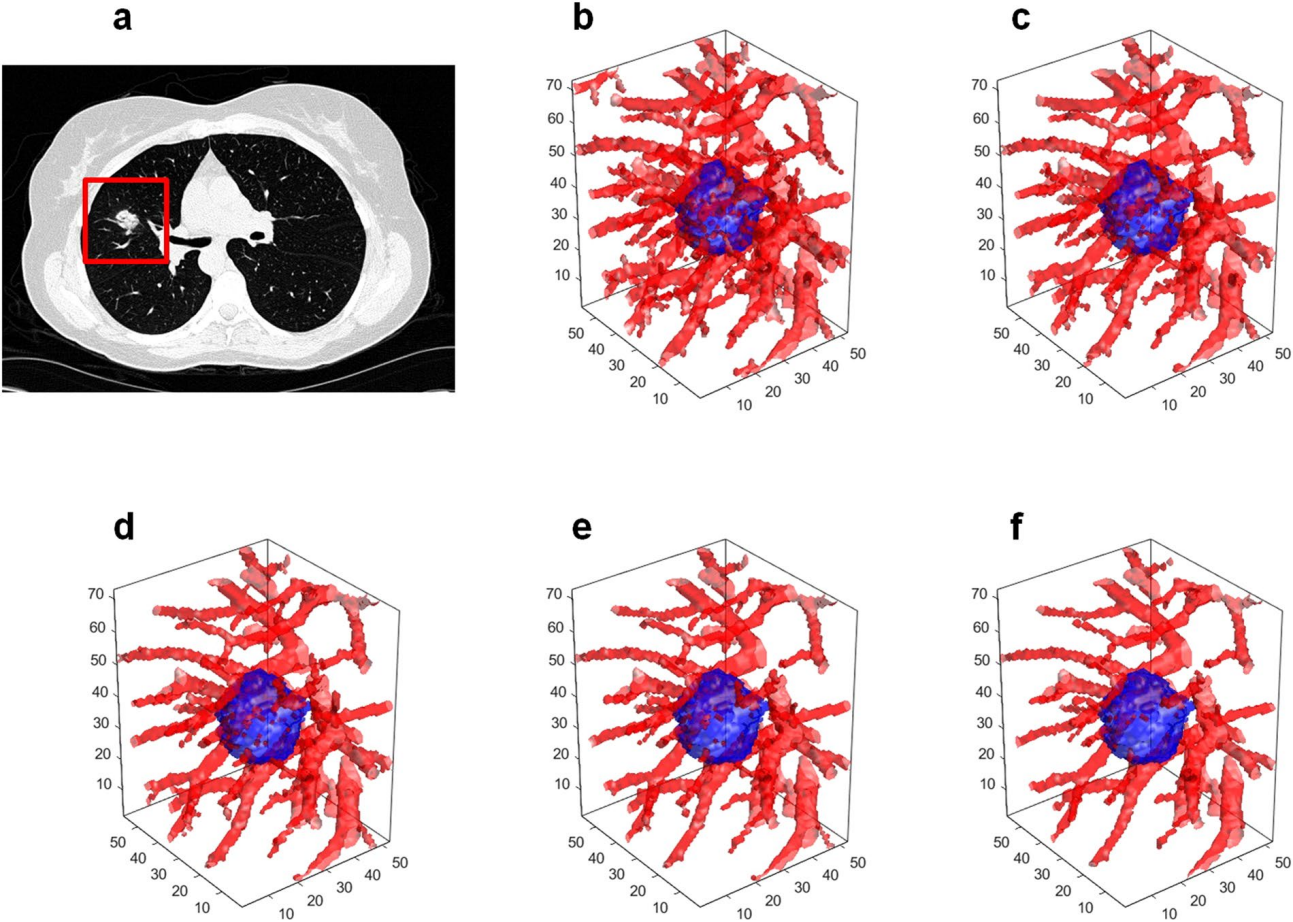
(**a**) A nodule and the region of interest. (**b**–**f**) Segmentations of the same nodule and its corresponding vasculature which were generated by assigning the following values respectively to the parameter *S*: −150, −100, −50, 0 and 50.

This section presents the sensitivity of QVT features to minor changes in the vasculature segmentation. We identified the parameter *S* which controls the growing of the vasculature volume during the segmentation process. This parameter corresponds to the mean difference value in Hounsfield units between a growing volume and the candidate voxels in the neighborhood of the vasculature. The values of *S* were identified empirically *S* ∈ {−150, −100, −50, 0, 50}. For each of the values in *S*, we generated multiple segmentations for each nodule and its surrounding vasculature on the CT scan. Figure 10 (b,c,d,e,f) illustrates 5 segmentations of a nodule and its corresponding vasculature, see Fig. 10(a). Segmentations were generated based on the values of *S*. Then the QVT features were calculated for all 5 versions of segmentations. The QVT features were calculated for all of the training set examples. We denote the QVT features corresponding to each of the *S* values as *QVT*_−150_, *QVT*_−100_, *QVT*_−50_, *QVT*_0_ and *QVT*_50_ respectively. To measure sensitivity of QVT features to slight changes in vessel segmentation, we computed the correlation between QVT pairs which were denoted by *Corr*(*QVT*_*s*1_, *QVT*_*s*2_). Some features (13,14, 15, 16) were highly stable and correlated across changes in vessel segmentation. However, some features (17, 18, 19, 20) were found to show more abrupt changes on account of minor perturbations in vessel segmentation. Consequently, these features were not highly correlated between different segmentations. The AUC values of the *C*_*QVT*_ classifier for each *QVT*_*S*_ were computed.

As shown in Table 7, the *C*_*QVT*_ classifier yielded an AUC = 0.79 ± 0.06,0.82 ± 0.12,0.87 ± 0.11,0.75 ± 0.14 and 0.69 ± 18, respectively for the different values of *S*. A more detailed description of these findings is provided in section G of the Supplemental Material.

**Table 7:** AUC values of the *C*_*QVT*_ classifier as a function of parameter *S*.

Features	*QVT*_−150_	*QVT*_−100_	*QVT*_−50_	*QVT*_0_	*QVT*_50_
AUC	0.79 ± 0.06	0.82 ± 0.12	0.87 ± 0.11	0.75 ± 0.14	0.69 ± 18

### Feature extraction

We extracted 35 QVT features from the nodule vasculature in each CT scan. The extracted QVT features are based on three important properties of the vessels: (I) tortuosity, (II) curvature and (III) branching statistics and the volume measurements of the vasculature. A detailed description of the QVT features is presented on Table S1 of the Supplementary Document. The QVT features attempt to quantify convolutedness and tortuosity of the nodule vasculature. Following segmentation of the nodule vasculature from the surroundings, the fast marching algorithm^58^ is employed to extract the skeleton of the vasculature. The nodule vasculature is assumed to be comprised of several vessel branches where each branch in turn is comprised of several points in a 3D space. The QVT features were measured for points, vessel branches and the entire vasculature. The torsion of a branch is defined as the ratio of the Euclidean distance between the starting and end points of a branch to the length of the branch. The torsion of a whole vasculature, is then calculated by computing the first order statistics of the torsion measurements associated with the branches of a vasculature.

The curvature is computed of points, branches and the entire vasculature. The curvature of a point which is located on the center line of a vessel in a 3D space is defined as the inverse of the radius of an osculating circle fitted to that point and also its left and right neighbors. The statistical moments of the curvature measurements of the points associated with the branches were computed to describe the curvature of a branch and similarly the curvature of the entire vasculature. In addition to torsion and curvature, the branching statistics of the vasculature were computed as well. These measurements refer to the number of small vessel branches in the vasculature. Feature extraction algorithms were implemented in MATLAB 2014b platform (Mathworks, Natick, MA).

### Data analysis

A data analysis pipeline was designed to evaluate the discriminability of the QVT features. The data analysis pipeline comprised of the following steps: a) Identifying the most discriminating QVT features and cluster analysis, b) stability analysis of the QVT features identified as most discriminating, c) Evaluating the ability of the QVT features to distinguish adenocarcinomas from granulomas, d) Comparing QVT with the state of the art radiomic features, e) Quantitative comparison of the discriminability of the QVT features compared to the corresponding diagnosis of two expert human readers on the validation set f) Statistical analysis between patients and CT specific parameters with histopathologic diagnosis of the nodule. The following sections provide the details pertaining to these various individual steps.

#### Identifying the most discriminating QVT features and cluster analysis

The mRMR^30^ feature selection algorithm was employed to identify the most predictive of the 35 QVT features from among the cases in *D*_1_. The AUC value for each of the most predictive QVT features was calculated.

An unsupervised cluster analysis based off the mRMR identified subset of QVT features was also performed on the cases in *D*_1_. The purpose of unsupervised clustering was to provide an alternative evaluation of the QVT features in addition to supervised classification. We also sought to discover patient clusters that showed corroboration between the QVT features and clinical parameters. A k-means clustering^60^ algorithm was applied to the QVT and clinical parameters for all patients in *D*_1_. To achieve this, unsupervised clustering of the patients into two clusters was carried out based off the top 12 QVT features identified via mRMR. Then, potential association of the predicted cluster numbers with the clinical data (i.e, histopathologic diagnosis of the nodules) is determined by the *χ*^2^ test which is used to determine whether a significant association between the expected and the observed frequencies in one or more categories exists^61^. To identify the most stable and dominant clusters of the patients we also performed unsupervised ensemble clustering on the QVT features for the patients in *D*_1_. This approach involved clustering the patients several times with different clustering parameters such as cluster number and distance metric. Next, we counted the co-occurrence of the pairs of samples, falling into the same cluster. Then the emerging clusters were compared with the corresponding clinical information for the patients.

#### Stability analysis of the QVT features identified as most discriminating

Stability is an important consideration in assessing the performance of radiomic features^62^. One specific attribute desireable in radiomic features is that the feature expression should either not change (or minimally change) for test-retest scans acquired within a short interval duration^63^. Since images obtained across different CT scanners, institutions, sites, vendors and acquisition parameters (e.g. reconstruction slice thickness, contrast enhancement and convolutional kernel) tend to vary in appearance, it is critical to assess the ability of radiomic features to deal with these variations. Additionally image variations can also result temporally in a single site due to scanning position and patient specific attributes. Unstable radiomic features, even ones identified as highly predictive on a training set, could yield a markedly inferior classification performance on an independent test set. To assess the stability of the QVT features, we used the independent reference imaging database to evaluate response (RIDER)^64^ lung cancer dataset which consists of same-day repeated test and re-test CT scans for 31 patients^65^. The intraclass correlation coefficient (ICC) was used to assess the stability of the top QVT features identified via the mRMR feature selection approach. ICC varies between −1 to 1, where ICC = 1 corresponds to a highly reproducible feature and ICC = 0 corresponds to a feature which is not highly reproducible and hence unstable.

#### Evaluating the ability of the QVT features to distinguish adenocarcinomas from granulomas

The set of QVT features identified as stable and discriminating were used to train supervised machine learning algorithms to predict the risk of malignancy of nodules on CT scans. The classifiers included a SVM^66^, Naive Bayes^67^ and KNN^68^ classifier. All classifiers were trained using the instances in the learning set (*D*_1_) and then validated on the independent test set (*D*_2_). For each of these classifiers, training was performed using a 3-fold cross validation re-sampling technique on *D*_1_. The best performing classifier (*C*_*QVT*_) was selected based on the computed AUC value for discriminating adenocarcinomas and granulomas in *D*_1_. The best performing classifier was then locked down and *C*_*QVT*_ was evaluated in terms of classification performance on the independent validation set (*D*_2_).

#### Comparison with state of the art radiomic features

The performance of *C*_*QVT*_ was compared with established radiomic features pertaining to nodule texture and shape (*C*_*rad*_). In this regard, a total of 669 well-known radiomic features including 645 2D texture and intensity, along with 24 3D shape features were extracted from the segmented nodule on the CT scan. All features were extracted in 3D. The texture features comprised local binary patterns, gradient, Gabor filter features, Laws-Laplacian pyramidal features, Law and Haralick features^69,70^. Shape features aimed at capturing irregularities and spiculations of nodule shape. Additionally these features also included measurements relating to geometrical properties of the nodule including size, compactness, eccentricity, elongation, convexity and sphericity. The description of these comparative radiomic features can be found in Tables S2 and S3 of the Supplementary Information file.

#### Comparison with deep learning networks

The performance of *C*_*QVT*_ was compared against two deep learning classifiers (*C*_*LeNet*_, *C*_*VGGNet*_). Two deep networks including a simple LeNet architecture and a VGG-19 CNN, in a transfer learning set up, were used for this purpose. The LeNet architecture comprised two sets of convolutional, rectified linear unit (ReLU) activation, and pooling layers, followed by a fully-connected layer, activation, another fully-connected, and finally a softmax classifier. For LeNet architecture,we used a simple patch-based classification approach, the softmax classifier returns the probability of each patch belonging to the two classes of interest. The model was trained over 100 epochs after which the weights were locked down. The performance metric across the training runs are shown in section I of the Supplemental Material. The learned weights were then evaluated on the independent validation set of 145 studies, and the predicted probabilities were utilized to generate the receiver operating characteristic curve. The VGG-19 CNN which was previously trained on the ImageNet database, was used as a feature extractor. The extracted features were then used to build a classifier on the training set, using a Random Forest classifier, and then evaluated on the test set.

#### Comparison with human experts

The classification performance of *C*_*QVT*_ was compared against the nodule diagnosis of two human experts on the cases in *D*_2_. A board certified attending radiologist with 10 years of experience in thoracic radiology and a pulmonologist with 4 years of experience in reading chest CT scans served as Readers 1 and 2 respectively. Both readers were blinded to the true histopathologic diagnosis of the 145 cases which comprised *D*_2_. Each reader was asked to assign a score between 1 to 5 to each nodule, with 1 referring to a high confidence that the nodule is “benign”, 2 referring to a diagnosis of “mostly benign”, 3 being “not sure”, 4 being “mostly malignant”, and 5 being “malignant”. To evaluate the performance of the experts, the classifier probability output was compared to diagnostic ground truth determined from the pathology reports. Based off the interpretations of the individual human readers, a ROC curve was obtained and the corresponding AUC was calculated.

#### Statistical analysis between patient and CT specific parameters with histopathologic diagnosis of the nodule

Statistical significance test was carried out between patient and CT specific parameters against histopathologic diagnosis of the nodule. The p-values were computed using the two tailed and unpaired Student t-test for continuous variables and Fishers exact test for categorical data. The threshold for determining statistical significance was considered by p < 0.01. The purpose of t-test analysis was to demonstrate that no significant difference existed in values for a parameter (nodule size for example) between the adenocarcinoma and granuloma groups in *D*_1_. Similarly, the purpose of the Fisher’s exact test was to show that there was no significant association between a categorical parameter (gender for example) and disease outcome. Patient and CT parameters included ‘Gender’, ‘Smoking status’, ‘Ethnicity’, ‘Age’, ‘Nodule size’ and ‘Slice thickness’.

#### Comparing nodule adjacent QVT with normal lung QVT

The QVT features from both non-healthy (nodule and its surrounding vasculature) and healthy regions (non-tumor) of the lung were extracted from both adenocarcinoma and granuloma cases. For non-healthy regions, we considered a healthy region with the same size corresponding to the opposite lung. The position of a healthy region on the opposite lung was determined according to the line of symmetry between the left and right lungs (see Fig. 6a). The healthy regions obtained from the normal lung opposite to the adenocarcinomas are referred to as Healthy_A and similarly healthy regions obtained from the normal lung opposite to the granulomas are referred to as Healthy_G. Next, QVT features were extracted from both non-healthy and healthy regions and the top ranked QVT feature was compared between these two regions using the paired student t-test. Non-healthy regions included adenocarcinomas and granulomas while healthy regions included the Healthy_A and Healthy_G.

## Electronic supplementary material

supplementary material
