# Author Correction: Quantitative vessel tortuosity: A potential CT imaging biomarker for distinguishing lung granulomas from adenocarcinomas

**DOI:** 10.1038/s41598-019-52008-9

**Published:** 2019-10-29

**Authors:** Mehdi Alilou, Mahdi Orooji, Niha Beig, Prateek Prasanna, Prabhakar Rajiah, Christopher Donatelli, Vamsidhar Velcheti, Sagar Rakshit, Michael Yang, Frank Jacono, Robert Gilkeson, Philip Linden, Anant Madabhushi

**Affiliations:** 1 Department of Biomedical Engineering, Case Western Reserve University, Cleveland, OH 44106 USA; 2 University Hospital Case Medical Center, Cleveland, OH 44106 USA; 3 Taussig Cancer Institute, Cleveland Clinic, Cleveland, OH 44106 USA; 4 Louis Stokes Cleveland VA Medical Center, Cleveland, OH 44106 USA

Correction to: *Scientific Reports* 10.1038/s41598-018-33473-0, published online 16 October 2018

This Article contains an error in the Discussion.

The text,

“Nearly all of the positive results were negative for cancer, producing a false-positive rate of 97.5% from radiologist based CT interpretations of the CT exams.”

should read:

“Nearly all of the positive results were negative for cancer, producing a high proportion of false positives within the positive results (97.5%) for lung cancer, from radiologist based CT interpretations of the CT exams.”

