# Supplementary material for: Quantitative vessel tortuosity: A potential CT imaging biomarker for distinguishing lung granulomas from adenocarcinomas
Source: Sci Rep. 2018 Oct 16;8:15290. doi: 10.1038/s41598-018-33473-0 (PMC6191462; doi:10.1038/s41598-018-33473-0)
Supplement: Supplementary file 1 — supplementary material [file 41598_2018_33473_MOESM1_ESM.pdf]

Supplementary Information

for

"Quantitative vessel tortuosity: A potential CT imaging biomarker for distinguishing lung granulomas from adenocarcinomas"

Mehdi Alilou[1*], Mahdi Orooji[1], Niha Beig[1], Prateek Prasanna[1], Prabhakar Rajiah[2], Christopher Donatelli[2], Vamsidhar Velcheti[3], Sagar Rakshit[3], Michael Yang[2], Frank Jacono[4], Robert Gilkeson[2], Philip Linden[2] & Anant Madabhushi[1]

[1]Department of Biomedical Engineering, Case Western Reserve University, Cleveland, OH 44106, USA
[2]University Hospital Case Medical Center, Cleveland, OH 44106, USA
[3]Taussig Cancer Institute, Cleveland Clinic, Cleveland, OH 44106, USA
[4]Louis Stokes Cleveland VA Medical Center, Cleveland, OH 44106, USA

A. Extraction of QVT features:

We extracted 35 QVT features from the nodule vasculature in each CT scan. The extracted QVT features are based on three important properties of the vessels: (I) tortuosity, (II) curvature and (III) branching statistics and the volume measurements of the vasculature. The nodule vasculature is assumed to be comprised of several vessel branches where each branch in turn is comprised of several points in a 3D space. The QVT features were derived from measurements relating to points, vessel branches and the entire vasculature.

The curvature of a point which is located on the center line of a vessel in 3D space is defined as the inverse of the radius of an osculating circle fitted to that point with respect to its immediate neighbors. The statistical moments of the curvature measurements of the points associated with the branches are computed to describe the curvature of a branch and similarly the curvature of the entire vasculature.

The torsion of a branch is defined as the ratio of the Euclidean distance between the starting and end points of a branch to the length of the branch. The torsion of a whole vasculature, is then calculated by computing the first order statistics of the torsion measurements associated with the branches of a vasculature. In addition to torsion and curvature, the branching statistics of the vasculature was computed as well. These measurements refer to the number of small vessel branches in the vasculature.

Each nodule vasculature is comprised of several vessel branches as illustrated in Fig.S1(a) and Fig.S1(b), where each vessel branch in turn is comprised of several points in 3D space (Fig.S2(a)).

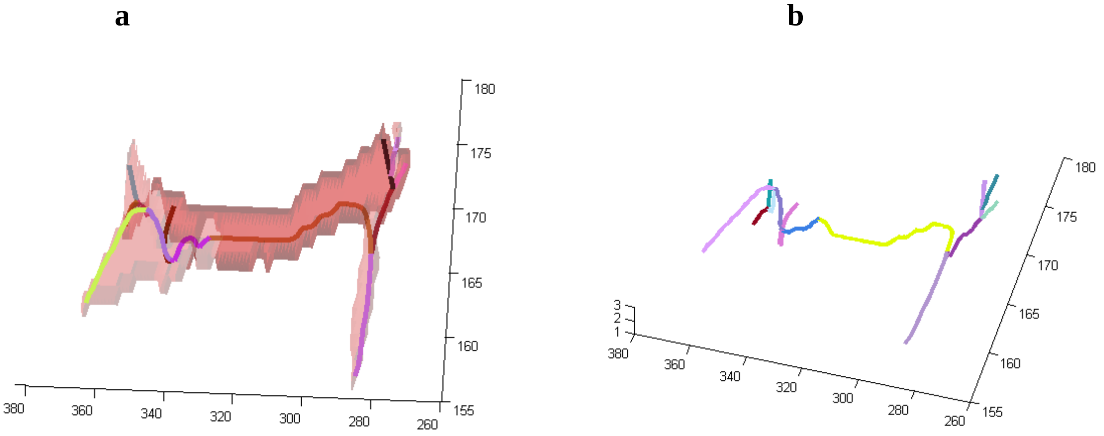

Fig.S1. a) A vasculature and its associated vessel branches, b) constituent segments of the branches of the same vasculature.

a **b**

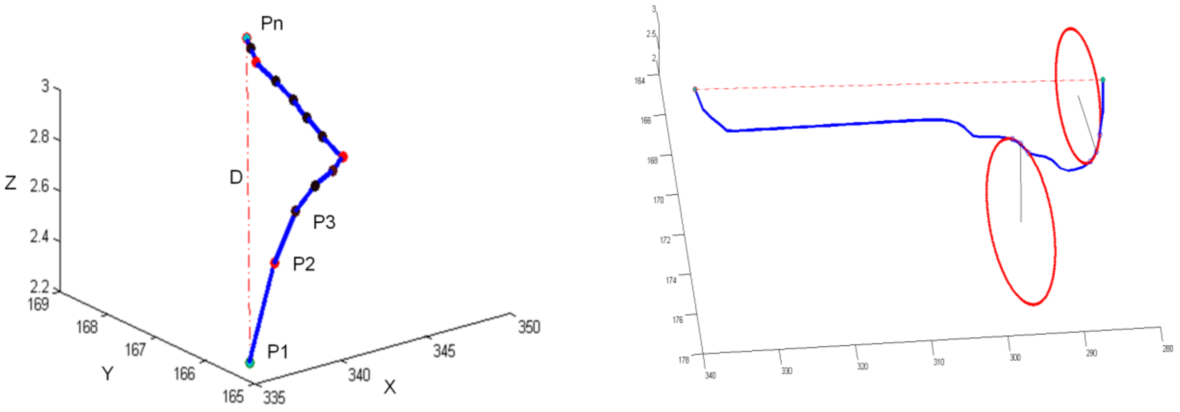

Fig.S2 a) A skeleton of a vessel branch and its constituent 3D points, b) To measure the curvature, the radius of an osculating circle is calculated for each point on a vessel branch.

Notations:

Notation	***Definition***
V	Nodule vasculature which is comprised of several branches. $V = \bigcup_{i=1}^{m} C_i$
C_i	A curve corresponding to the center line of a vessel branch in 3D. $C_i = \bigcup_{j=1}^{n} P_j$
P_j	A 3D point on a C_i with coordinates of (x_j, y_j, z_j)
$\lvert P_{j-1} - P_j \rvert$	Euclidean distance between P_{j-1} and P_j
L_{c_i}	Length of a C_i
D_{c_i}	Euclidean distance between the first (P_1) and the last (P_n) points of *a* C_i
T_{c_i}	Torsion of C_i
k_{p_j}	Curvature of a point P_j
k_{c_i}	Curvature of a vessel branch C_i

Formulations:

Assume that the vasculature (V) for each nodule is comprised of branches which were converted to its corresponding skeleton in the form of a line (C_i) in 3D space. We also assume that a 3D line (C_i) is in turn comprised of several points (P_j). We denote the Euclidean distance between two immediate close points (P_{j-1}, P_j) on C_i by $\lvert P_{j-1} - P_j \rvert$ and hence the length of a vessel branch (L_{c_i}) is then approximated as follows,

$$L_{c_i} \approx \sum_{j=1}^{n} |P_{j-1} - P_j|,$$

where n is the number of points on a vessel branch and $|P_{j-1} - P_j|$ is computed as follows:

$$|P_{j-1} - P_j| = \sqrt{(x_j - x_{j-1})^2 + (y_j - y_{j-1})^2 + (z_j - z_{j-1})^2}.$$

Where $(x_{j-1}, y_{j-1}, z_{j-1})$ and (x_j, y_j, z_j) correspond to the coordinates of P_{j-1} and P_j respectively.

We also compute the direct distance between P_1 and P_n which corresponds to the length of D_{c_i} in Fig.S2(a) as follows:

$$D_{c_i} = |P_1 - P_n|.$$

Hence, the torsion of a branch is defined as:

$$T_{c_i} = 1 - \frac{D_{c_i}}{L_{c_i}}.$$

The torsion of nodule vasculature (V), is then calculated by computing the first order statistics of the torsion measurements (T_{c_i}) associated with the branches of a vasculature.

To obtain the curvature of a vessel branch, first, we calculate the curvature of its constituent points (see Fig2.b). Let $\vec{r}(t) = (x(t), y(t), z(t))$ be a vector-valued function of parameter t that models a smooth vessel branch curve C_i and let P be the point on C_i at t. The function $\vec{r}$ produces a point P on C_i by taking an input value t. The curvature at P_j is proportional to the inverse of the radius of an osculating circle at that point. The osculating circle at P_j is the circle that best approximates C_i at P_j. The radius (ρ) of the osculating circle at P_j is defined as:

$$\rho(t) = \frac{1}{k_{p_j}(t)},$$

where t is time parameter and $k_{p_j}(t)$ is the curvature at point P_j and is obtained from the following formula:

$$k_{p_j}(t) = \frac{\left(\left(\frac{d^2}{dt^2}z(t).\frac{d}{dt}y(t) - \frac{d^2}{dt^2}y(t).\frac{d}{dt}z(t)\right)^2 + \left(\frac{d^2}{dt^2}x(t).\frac{d}{dt}z(t) - \frac{d^2}{dt^2}z(t).\frac{d}{dt}x(t)\right)^2 + \left(\frac{d^2}{dt^2}y(t).\frac{d}{dt}x(t) - \frac{d^2}{dt^2}x(t).\frac{d}{dt}y(t)\right)^2\right)^{\frac{1}{2}}}{\left(\frac{d}{dt}x(t)^2 + \frac{d}{dt}y(t)^2 + \frac{d}{dt}z(t)^2\right)^{\frac{3}{2}}}$$

where $\frac{d}{dt}$ and $\frac{d^2}{dt^2}$ denote the first and second order derivative with respect to the parameter t and $x(t), y(t)$ and $z(t)$ are coordinate functions of the parameter t.

For k_{p_j} at each point $P_j \in C_i$, we calculate *Min*, *Max*, *Mean* and *Standard deviation (Std)* of k_{p_j} values to describe the curvature of a vessel branch (k_{c_i}). Then, the curvature of the whole vasculature (V) is acquired by computing the first order statistics of the aforementioned measurements associated with the branches of a vasculature. Table.S1 illustrates the description of QVT features.

Table S1: The QVT features and their description.

Features	Description
Torsion (f1, f2, f3)	*Mean*, *Std* and *Max* values of the branches torsion T_{c_i} in nodule vasculature *V*
Statistics of standard deviation of branches curvature (f4, f5, f6)	*Mean*, *Std* and *Max* values of the standard deviation of the branches curvature k_{c_i} in nodule vasculature *V*
Statistics of average curvature of the branches (f7, f8, f9)	*Mean*, *Std* and *Max* values of the averaged curvature of the branches k_{c_i}associated with vasculature *V*
Statistics of maximum curvature of the branches (f10, f11, f12)	*Mean*, *Std* and *Max* of the maximum curvature values of the branches k_{c_i} associated with vasculature *V*
Branching count of a vasculature (f13)	Number of branches C_i associated with a vasculature *V*
Normalized volume (f14)	Volume of a vasculature *V* divided by the volume of its bounding box
Volume (f15)	Volume of a vasculature *V*
Histogram of torsion (f16-f25)	Histogram of torsion measurements of the branches T_{c_i}in *V*
Histogram of curvature (f26-f35)	Histogram of curvature measurements of points k_{p_j}in *V*

* The top 12 most predictive QVT features were identified as the following features: f7, f22, f15, f10, f6, f29, f8, f35, f12, f11, f14, f34. The p-values of the top features were computed under the null hypothesis that there was no significant difference between the 12 QVT features between adenocarcinomas and granulomas were found to be : $1.5e^{-5}$, 0.18, 0.001, $8.7e^{-6}$, 0.01 , 0.03 , $6.04e^{-5}$, 0.004, $7.8e^{-5}$, 0.001, 0.0007, 0.09).

B. Well-known Texture and Shape Radiomics:

Table S2: Texture features evaluated in this work.

Feature category	Descriptor	Intuitive Description
Haralick features (Repeated occurrence of grey level configuration in the texture represented via the grey-level co-occurrence matrix (GLCM), which varies rapidly with distance in fine textures and slowly in large textures)	Inverse Difference Moment (IDM)	IDM is a reflection of the presence or absence of uniformity, and hence is a measure of local regions of homogeneity<br>High IDM: Higher presence of locally uniform windows in GLCM<br>Low IDM: Higher presence of locally heterogeneous windows in GLCM
	Correlation	Quantifies the linear patterns in an image based on the distance parameter.
	Sum Entropy	Measure of GLCM relationship to distribution of intensity with respect to entropy. Entropy is the measure of disorder.
	Sum Variance	Measure of GLCM relationship to distribution of intensity with respect to variance<br>High sum variance: greater standard deviation of sum average<br>Low sum variance: low standard deviation of sum average
Laws features	E5, L5, S5,W5,R5 (combination in both *X* and *Y* directions)	E- Edges<br>L- Level<br>S- Spots<br>W- Wave<br>R- Ripple
Laplacian pyramids		Multi-resolution filters capture edges at different levels
Gray level features		The basic, intensity based features including mean, median, range and standard deviation.
Gabor Features		Oriented textures via changes in direction and scale; capture microarchitectures
Gradient Features		Represent the directional change in the intensity values of pixels in the ROI
Local Binary Pattern		Thresholding the window with the center pixel value.

Table S3: Shape features evaluated in this work.

Features	Description
Size	Including Width, Height, Depth of bounding box
Area	from 2D slices of each nodule
Perimeter	from 2D slices of each nodule
Eccentricity	Distance between the foci of the ellipse and major axis length
Extend	ratio of pixels in the region to pixels in the total bounding box
Compactness	ratio of the perimeter squared to the product of 4π and area
Radial distance	Mean distance from center of each slice to contour points
Roughness	perimeter of slices divided by convex perimeter
Elongation	from major and minor axis
Convexity	from convex hull
Equivalent Diameter	Diameter of circle with same area of slices
Sphericity	3D compactness

C. Human-Machine comparison:

Table S4: Human-Machine (SVM) comparison results on the test set (n=145). AUC values were generated for each of the human readers as well as the machine classifier (SVM). A hard decision was obtained for each case to be a granuloma by using a threshold of <3 and >50% respectively for the human readers and the machine classifier. Each green cell represents a decision either by the machine classifier or the human reader when the hard decision matched the true pathologic diagnosis for that nodule and pink when it did not match.

Case name	***Ground Truth***	***Blind name***	***Reader1 Score***	***Reader2 Score***	***Machine Score***	***Reader1 Decision***	***Reader2 Decision***	***Machine Decision***
AAA344A	Adeno	131	4	4	**5**	'Adeno'	'Adeno'	'Adeno'
A 3000	Adeno	76	4	4	**5**	'Adeno'	'Adeno'	'Adeno'
A 3001	Adeno	49	3	3	**5**	'grano'	'grano'	'Adeno'
A 3002	Adeno	51	4	4	**5**	'Adeno'	'Adeno'	'Adeno'
A 3004	Adeno	47	3	3	**5**	'grano'	'grano'	'Adeno'
A 3005	Adeno	53	4	3	**4**	'Adeno'	'grano'	'Adeno'
A 3007	Adeno	40	3	3	**4**	'grano'	'grano'	'Adeno'
A 3008	Adeno	41	3	2	**4**	'grano'	'grano'	'Adeno'
A 3011	Adeno	42	3	3	**1**	'grano'	'grano'	'Grano'
A 3013	Adeno	103	3	3	**5**	'grano'	'grano'	'Adeno'
A 3015	Adeno	5	4	2	**4**	'Adeno'	'grano'	'Adeno'
A 3016	Adeno	67	4	4	**4**	'Adeno'	'Adeno'	'Adeno'
A 3017	Adeno	68	3	3	**1**	'grano'	'grano'	'Grano'
A 3018	Adeno	46	4	4	**5**	'Adeno'	'Adeno'	'Adeno'
A 3020	Adeno	78	3	2	**5**	'grano'	'grano'	'Adeno'
A 3021	Adeno	84	3	3	**5**	'grano'	'grano'	'Adeno'
A 3022	Adeno	80	3	3	**5**	'grano'	'grano'	'Adeno'
A 3023	Adeno	69	4	3	**5**	'Adeno'	'grano'	'Adeno'
A 3040	Adeno	70	4	3	**5**	'Adeno'	'grano'	'Adeno'
A 3042	Adeno	71	2	2	**2**	'grano'	'grano'	'Grano'
A 3043	Adeno	72	3	3	**5**	'grano'	'grano'	'Adeno'
A 3045	Adeno	73	5	3	**5**	'Adeno'	'grano'	'Adeno'
A 3046	Adeno	104	3	2	**5**	'grano'	'grano'	'Adeno'
A 3047	Adeno	105	4	4	**1**	'Adeno'	'Adeno'	'Grano'
A 3048	Adeno	56	3	3	**2**	'grano'	'grano'	'Grano'
A 3049	Adeno	57	3	3	**2**	'grano'	'grano'	'Grano'
A 3050	Adeno	99	3	2	**1**	'grano'	'grano'	'Grano'
A ggg12A	Adeno	10	4	4	**1**	'Adeno'	'Adeno'	'Grano'
AAA 578A-N2	Adeno	97	4	4	**1**	'Adeno'	'Adeno'	'Grano'

AAA126A	Adeno	90	4	3	2	'Adeno'	'grano'	'Grano'
AAA164A	Adeno	91	3	4	5	'grano'	'Adeno'	'Adeno'
AAA200A	Adeno	93	3	3	5	'grano'	'grano'	'Adeno'
AAA255A	Adeno	129	4	4	5	'Adeno'	'Adeno'	'Adeno'
AAA275A	Adeno	130	4	3	5	'Adeno'	'grano'	'Adeno'
AAA399A	Adeno	132	3	1	5	'grano'	'grano'	'Adeno'
AAA484A	Adeno	145	4	3	1	'Adeno'	'grano'	'Grano'
AAA492A	Adeno	94	4	4	2	'Adeno'	'Adeno'	'Grano'
AAA575A	Adeno	95	5	4	1	'Adeno'	'Adeno'	'Grano'
AAA578A -N1	Adeno	96	3	4	1	'grano'	'Adeno'	'Grano'
AAA594A	Adeno	98	4	5	1	'Adeno'	'Adeno'	'Grano'
AAA601A	Adeno	33	3	3	1	'grano'	'grano'	'Grano'
AAA854A	Adeno	34	4	5	1	'Adeno'	'Adeno'	'Grano'
AS1	Adeno	11	3	3	1	'grano'	'grano'	'Grano'
AS2	Adeno	12	3	4	1	'grano'	'Adeno'	'Grano'
AS3	Adeno	13	3	3	1	'grano'	'grano'	'Grano'
AS4	Adeno	58	2	3	1	'grano'	'grano'	'Grano'
AS5	Adeno	59	2	3	1	'grano'	'grano'	'Grano'
CL 3044A	Adeno	35	4	3	1	'Adeno'	'grano'	'Grano'
CL 3074B	Adeno	36	4	4	1	'Adeno'	'Adeno'	'Grano'
CL 3075B	Adeno	37	4	4	2	'Adeno'	'Adeno'	'Grano'
CL 3076B	Adeno	6	4	3	2	'Adeno'	'grano'	'Grano'
CL 3083B	Adeno	7	3	3	1	'grano'	'grano'	'Grano'
CL 3130	Adeno	8	2	3	5	'grano'	'grano'	'Adeno'
CL 3131B	Adeno	9	4	4	2	'Adeno'	'Adeno'	'Grano'
CL 3132A	Adeno	30	4	4	1	'Adeno'	'Adeno'	'Grano'
CL 3133A	Adeno	108	3	5	3	'grano'	'Adeno'	'Grano'
CL 3134	Adeno	110	3	4	2	'grano'	'Adeno'	'Grano'
CL 3135B	Adeno	137	4	3	1	'Adeno'	'grano'	'Grano'
CL 3136	Adeno	138	5	5	1	'Adeno'	'Adeno'	'Grano'
CL 3137	Adeno	139	3	3	1	'grano'	'grano'	'Grano'
CL 3138	Adeno	142	3	3	1	'grano'	'grano'	'Grano'
CL 3139	Adeno	143	4	5	1	'Adeno'	'Adeno'	'Grano'
CL 3140	Adeno	144	4	4	1	'Adeno'	'Adeno'	'Grano'
CL 3141	Adeno	112	2	3	1	'grano'	'grano'	'Grano'
CL 3142	Adeno	113	3	3	1	'grano'	'grano'	'Grano'
CL 3143	Adeno	114	5	5	5	'Adeno'	'Adeno'	'Adeno'
CL 3144	Adeno	115	3	2	5	'grano'	'grano'	'Adeno'
CL 3146	Adeno	116	4	4	5	'Adeno'	'Adeno'	'Adeno'
CL 3147	Adeno	117	4	4	1	'Adeno'	'Adeno'	'Grano'
CL 3148	Adeno	118	4	4	1	'Adeno'	'Adeno'	'Grano'

CL 3149	Adeno	119	4	3	**1**	'Adeno'	'grano'	'Grano'
CL 3150	Adeno	120	4	5	**1**	'Adeno'	'Adeno'	'Grano'
3	Grano	133	4	4	**1**	'Adeno'	'Adeno'	'Grano'
4	Grano	100	3	4	**1**	'grano'	'Adeno'	'Grano'
10	Grano	60	3	3	**1**	'grano'	'grano'	'Grano'
11	Grano	61	2	1	**3**	'grano'	'grano'	'Adeno'
20	Grano	62	2	3	**1**	'grano'	'grano'	'Grano'
31	Grano	63	2	3	**1**	'grano'	'grano'	'Grano'
45	Grano	64	2	2	**1**	'grano'	'grano'	'Grano'
50	Grano	65	3	3	**1**	'grano'	'grano'	'Grano'
52	Grano	66	2	2	**4**	'grano'	'grano'	'Adeno'
56	Grano	122	4	2	**1**	'Adeno'	'grano'	'Grano'
61	Grano	123	3	3	**1**	'grano'	'grano'	'Grano'
74	Grano	124	4	4	**1**	'Adeno'	'Adeno'	'Grano'
75	Grano	125	4	3	**1**	'Adeno'	'grano'	'Grano'
80	Grano	126	4	4	**1**	'Adeno'	'Adeno'	'Grano'
83	Grano	127	3	3	**1**	'grano'	'grano'	'Grano'
95	Grano	128	4	3	**1**	'Adeno'	'grano'	'Grano'
3052	Grano	134	2	4	**1**	'grano'	'Adeno'	'Grano'
3059	Grano	135	3	3	**1**	'grano'	'grano'	'Grano'
3061	Grano	136	2	4	**1**	'grano'	'Adeno'	'Grano'
3067	Grano	140	3	3	**1**	'grano'	'grano'	'Grano'
3069	Grano	141	4	4	**1**	'Adeno'	'Adeno'	'Grano'
3074	Grano	28	4	4	**1**	'Adeno'	'Adeno'	'Grano'
3076	Grano	29	4	3	**2**	'Adeno'	'grano'	'Grano'
3078	Grano	19	3	3	**2**	'grano'	'grano'	'Grano'
3079	Grano	20	2	1	**1**	'grano'	'grano'	'Grano'
3080	Grano	21	4	5	**1**	'Adeno'	'Adeno'	'Grano'
3081	Grano	22	4	3	**1**	'Adeno'	'grano'	'Grano'
3082	Grano	23	4	4	**1**	'Adeno'	'Adeno'	'Grano'
3084	Grano	24	3	3	**1**	'grano'	'grano'	'Grano'
3087	Grano	25	4	4	**3**	'Adeno'	'Adeno'	'Grano'
3088	Grano	15	3	2	**1**	'grano'	'grano'	'Grano'
3090	Grano	16	4	4	**1**	'Adeno'	'Adeno'	'Grano'
3093	Grano	17	4	3	**1**	'Adeno'	'grano'	'Grano'
3096	Grano	18	3	2	**1**	'grano'	'grano'	'Grano'
3097	Grano	38	3	3	**2**	'grano'	'grano'	'Grano'
3100	Grano	14	2	3	**1**	'grano'	'grano'	'Grano'
3105	Grano	31	2	2	**1**	'grano'	'grano'	'Grano'
3108	Grano	32	3	2	**1**	'grano'	'grano'	'Grano'
3110	Grano	121	2	2	**1**	'grano'	'grano'	'Grano'
CL 3024	Grano	39	3	2	**1**	'grano'	'grano'	'Grano'

CL 3025	Grano	44	3	3	**1**	'grano'	'grano'	'Grano'
CL 3026	Grano	45	3	3	**1**	'grano'	'grano'	'Grano'
CL 3027	Grano	50	2	4	**1**	'grano'	'Adeno'	'Grano'
CL 3028	Grano	26	3	3	**2**	'grano'	'grano'	'Grano'
CL 3030	Grano	27	3	2	**1**	'grano'	'grano'	'Grano'
CL 3031	Grano	101	2	1	**1**	'grano'	'grano'	'Grano'
CL 3032	Grano	85	3	3	**1**	'grano'	'grano'	'Grano'
CL 3033	Grano	86	4	4	**1**	'Adeno'	'Adeno'	'Grano'
CL 3034	Grano	92	4	3	**1**	'Adeno'	'grano'	'Grano'
CL 3035	Grano	87	4	3	**1**	'Adeno'	'grano'	'Grano'
CL 3036	Grano	52	2	2	**1**	'grano'	'grano'	'Grano'
CL 3037	Grano	111	4	5	**1**	'Adeno'	'Adeno'	'Grano'
CL 3038	Grano	88	4	4	**1**	'Adeno'	'Adeno'	'Grano'
CL 3051	Grano	89	2	1	**1**	'grano'	'grano'	'Grano'
CL 3053	Grano	77	3	3	**1**	'grano'	'grano'	'Grano'
CL 3054	Grano	102	2	3	**1**	'grano'	'grano'	'Grano'
CL 3055	Grano	54	4	3	**1**	'Adeno'	'grano'	'Grano'
CL 3056	Grano	55	3	2	**2**	'grano'	'grano'	'Grano'
CL 3057	Grano	48	3	1	**2**	'grano'	'grano'	'Grano'
CL 3060	Grano	81	4	4	**1**	'Adeno'	'Adeno'	'Grano'
CL 3062	Grano	82	4	3	**1**	'Adeno'	'grano'	'Grano'
CL 3063	Grano	83	3	5	**1**	'grano'	'Adeno'	'Grano'
CL 3064	Grano	107	1	1	**1**	'grano'	'grano'	'Grano'
CL 3065	Grano	106	3	3	**1**	'grano'	'grano'	'Grano'
CL 3066	Grano	109	4	3	**1**	'Adeno'	'grano'	'Grano'
CL 3068	Grano	79	4	4	**1**	'Adeno'	'Adeno'	'Grano'
CL 3070	Grano	43	4	4	**1**	'Adeno'	'Adeno'	'Grano'
CL 3071	Grano	1	4	4	**1**	'Adeno'	'Adeno'	'Grano'
CL 3072	Grano	2	4	4	**1**	'Adeno'	'Adeno'	'Grano'
CL 3073	Grano	3	3	3	**1**	'grano'	'grano'	'Grano'
CL 3074	Grano	4	4	3	**1**	'Adeno'	'grano'	'Grano'
CL 3077	Grano	74	3	2	**1**	'grano'	'grano'	'Grano'
CL 3078	Grano	75	3	3	**5**	'grano'	'grano'	'Adeno'

D. Inclusion and exclusion criteria of the data:

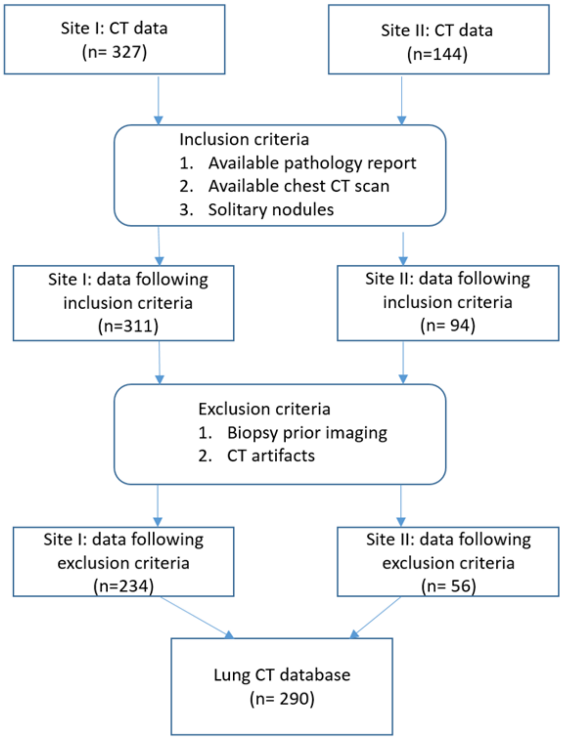

Fig.S3: The flowchart shows the inclusion and exclusion criteria for the patients' datasets employed in this study.

E. Comparison of feature selection strategies:

The AUC performance of the QVT features selected by mRMR, LASSO and PC as well as joint features selected by LASSO and mRMR are presented in Table.S5.

Table S5. The AUC performance of QVT features selected by mRMR, LASSO and PC on both training and validation sets.

Feature selection method	***Details***	***AUC on Training data***	***AUC on validation on test data***
mRMR	Top 12 features	0.93±0.03	0.86
LASSO	10 cross validations	0.93±0.02	0.78
PCA	3 principal components	0.92±0.04	0.72
mRMR & Lasso	8 joint features	0.91±0.03	0.82

Among the 12 features identified by mRMR, 8 features were also selected by LASSO. We believe that mRMR and LASSO are potentially more appropriate for the features considered in this study compared to PCA. This is potentially because PCA transforms the original features into a new feature space and uses a linear embedding of the high dimensional features which may actually lie on a non-linear manifold. Hence the linearity assumptions involved in the use of PCA may result in low dimensional embedding which may not be an accurate reflection of the structure of the original high dimensional feature space.

F. The impact of nodules position to the output of the method:

F.1. Association of the nodule location and diagnostic class:

To determine the association of a nodule's location with its corresponding diagnostic class, we captured the location information of each of the nodules. This included whether a nodule was located in the apical, mid or basal lung regions in 3D (see Fig.S4(b)). In the 2D transverse plane, we also identified whether a nodule was located within the central, upper or lower lung region (see Fig.S4(a)).

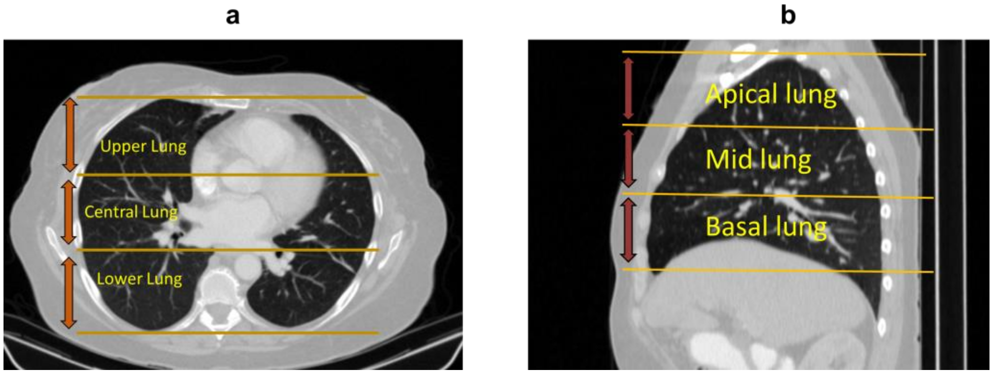

Figure S4. Upper, central and lower control regions on transverse (a) and sagittal (b) planes. The transverse plane was used to determine whether a nodule was close to the upper pleura, sub pleura or whether it was located in the central lung region. The sagittal plane was used to determine whether the nodule was located in the apical, mid or basal regions along z-axis.

Fig.S5 shows a frequency plot for the nodule position in both 3D and 2D for both adenocarcinomas and granulomas. Also we performed χ^2 test between nodule position and its class to discover possible dependency between these parameters. We have defined control positions and assigned 1, 2, and 3 respectively for nodules located in the upper, central and lower regions in 2D and 3D. The χ^2 test revealed that variables corresponding to both 2D position of the nodule in lung (Fig.S4-a) and 3D position (Fig.S4-b) were independent from nodules' diagnostic class. In other words, no significant association was identified between the position of the nodules and its corresponding diagnostic class (p>0.01). These results can be seen in Table S6.

Table S6. The p-values corresponding to the χ^2 test between the diagnostic class of a nodule and its relative position in 2D and 3D. p-value<0.01 was considered as indicating the presence of a significant association between a nodule's position and its diagnostic class.

	2D nodule position vs Nodule class				*nodule position along z-axis vs nodule class*			
	Training set		*Validation set*		*Training set*		*Validation set*	
p-value	*Left lung* ***0.05***	*Right lung* ***0.92***	*Left lung* ***0.49***	*Right lung* ***0.64***	*Left lung* ***0.06***	*Right lung* ***0.38***	*Left lung* ***0.75***	*Right lung* ***0.015***

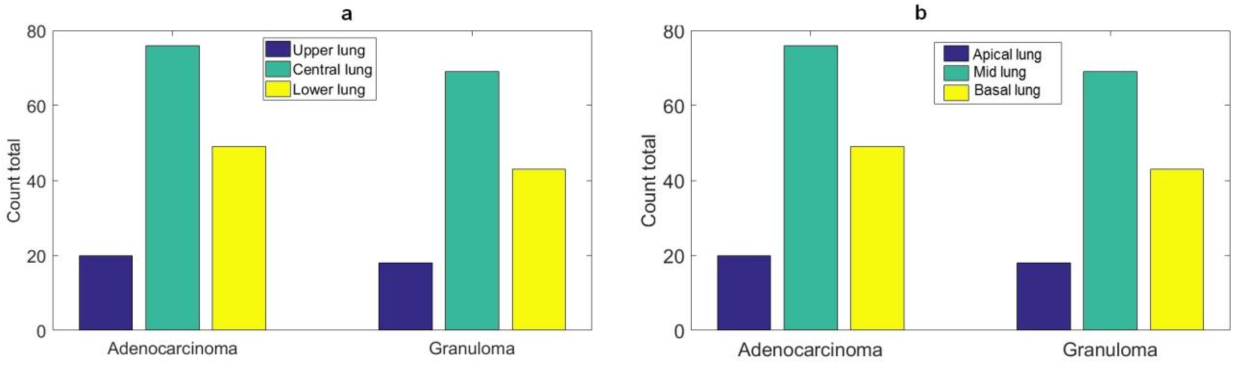

Figure S5. The distribution of the nodules based on their position in (a) the 2D transverse lung plane which include upper, central and lower lung nodules, (b) in the sagittal lung plane which include nodules located in the apical, mid and basal lung regions.

F.2. The impact of spatial location of a nodule on its corresponding QVT feature measurements:

In this section we evaluated to what extent the spatial location of a nodule impacts the corresponding QVT measurements of the nodules from the different diagnostic classes. First, we defined the following control regions to determine whether a nodule was located in the a) apical or lower lung portions, b) Gravity dependent (the lowest part of the lung in relation to gravity) or non-dependent portions of the lung and c) peripheral or central regions. Fig.S6 shows the control regions. Next, the QVT features for the 145 cases of the training set where extracted. Then for both adenocarcinomas and granulomas we determined whether the lung region impacted the QVT measurements. In this regard, an unpaired t-test was applied to the 10 top ranked QVT features of the nodules corresponding to the apical vs lower, dependent vs non-dependent and central vs peripheral lung regions. Fig.S7 shows the QVT features of the apical and lower lung adenocarcinomas. Similarly, Fig.S8 shows the QVT features corresponding to the apical and lower lung granulomas. As may be appreciated from figures S7-S8, QVT features were not found to be significantly different between apical and lower lung regions. As presented in figures S9 and S10, 1 QVT feature was found to be significantly different for the nodules located in the dependent and non-dependent lung regions. The statistically significantly different features were 1: *Mean* torsion of the vessel branches for adenocarcinomas, and 10: the ratio of vasculature volume to its bounding box for granulomas.

Finally, 5 QVT features (5: Branching count of the vasculature, 6: histogram of curvature, 7-10: torsion histogram) were significantly different between adenocarcinomas of central and peripheral regions. For the granulomas within the same regions, 3 QVT features (1,2: *Mean*, *Std* values of the torsion of the vessel branches, 10: histogram of torsion) were found to be significantly different (see Figs.S11-12).

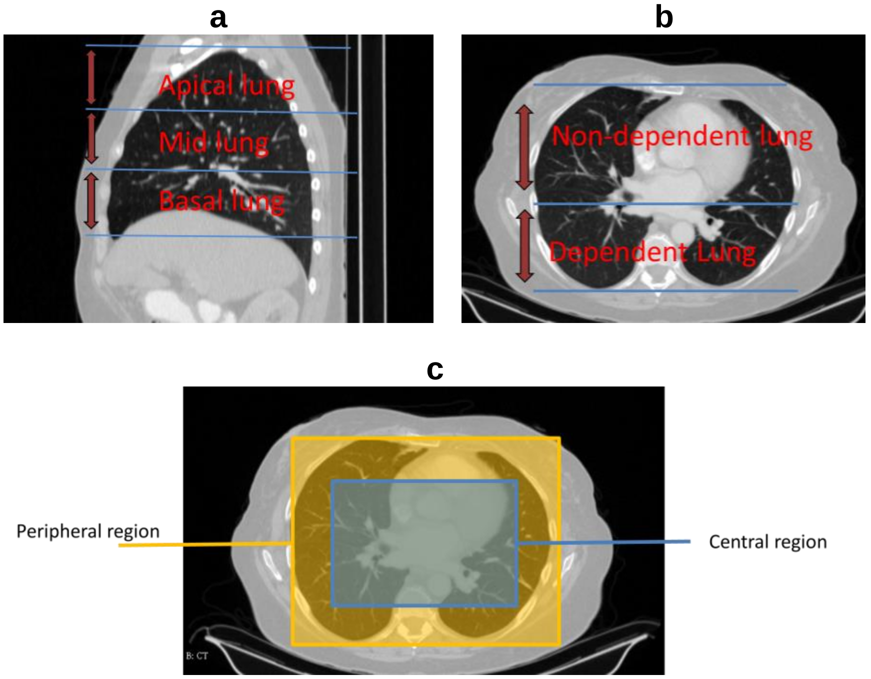

Figure S6. Control regions to determine whether a nodule belong to a) apical or lower lung portions, b) dependent or non-dependent lung and c) central or peripheral regions.

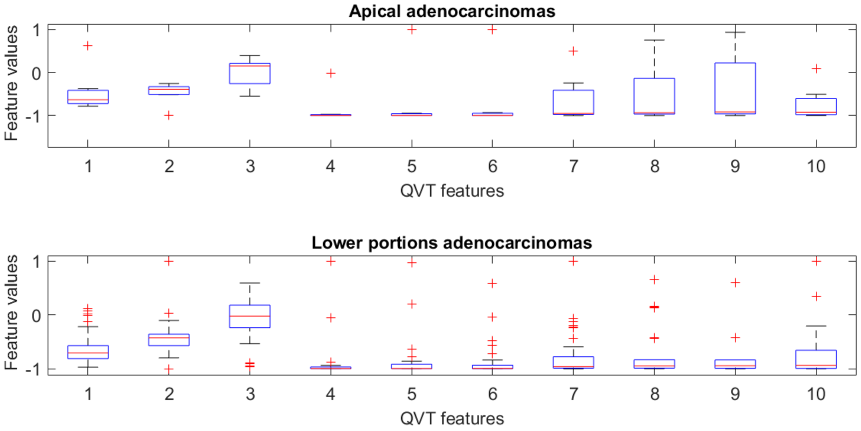

Figure S7. No significant differences were found between QVT features of the adenocarcinomas located in the apical and lower lung regions.

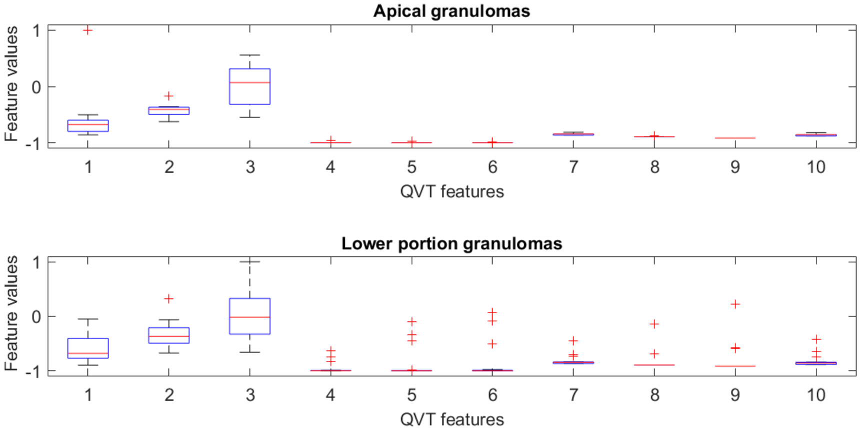

Figure S8. No significant differences were found between QVT features of the granulomas located in the apical and lower lung regions.

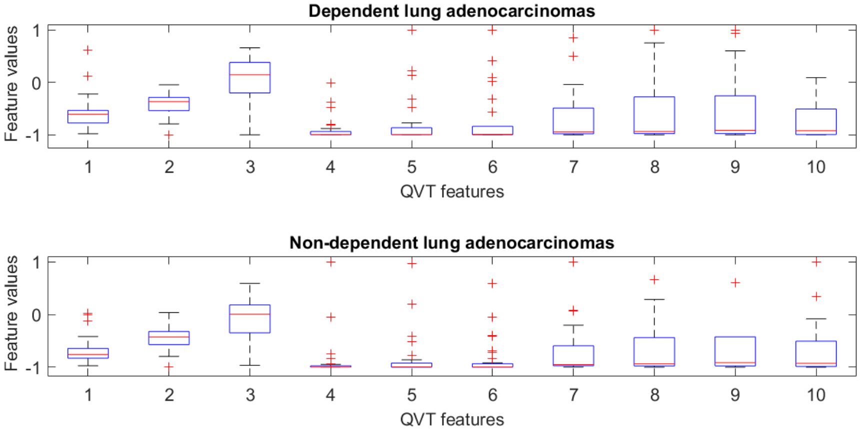

Figure S9. 1 QVT feature (1: *Mean* of the vessel branches' torsion) was significantly different between adenocarcinomas corresponding to regions from within the gravity dependent and non-dependent lung regions. The lowest part of the lung in relation to gravity was considered as the dependent region and the upper part was considered as non-dependent region.

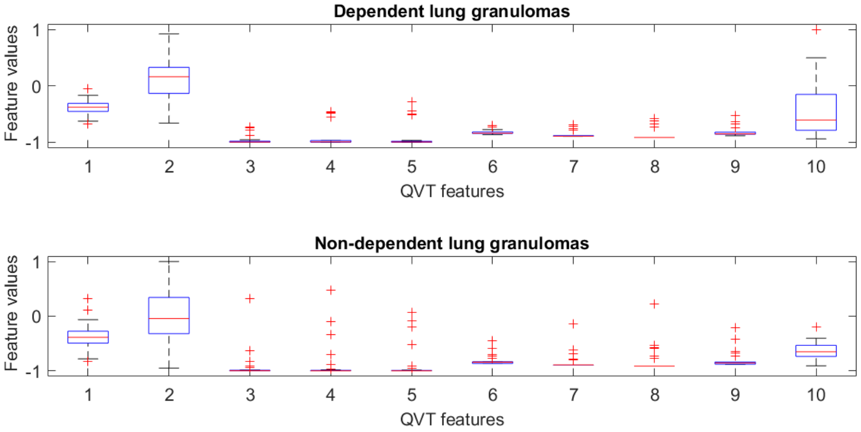

Figure S10. 1 QVT feature (10: the ratio of vasculature volume to its bounding box) was significantly different between granulomas corresponding to the regions from with gravity dependent and non-dependent lung. The lowest part of the lung in relation to gravity was considered as the dependent region and the upper part was considered as non-dependent region.

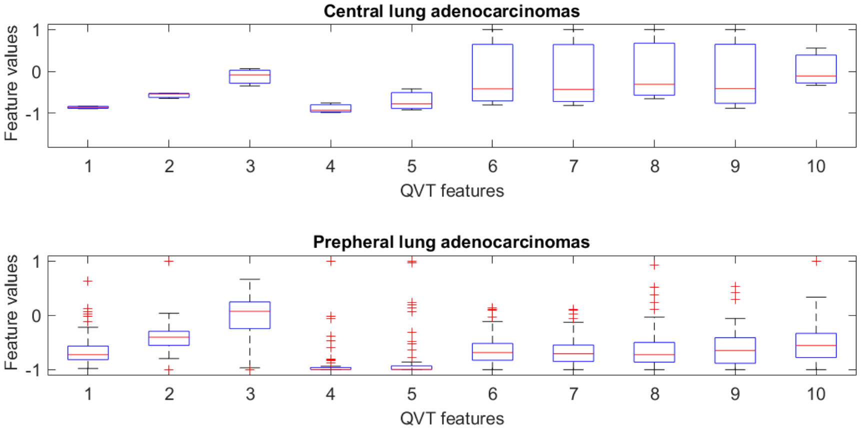

Figure S11. 5 QVT features (5: Branching count of the vasculature, 6: histogram of curvature, 7-10: torsion histogram) were significantly different between adenocarcinomas located within the central and peripheral regions of the lung.

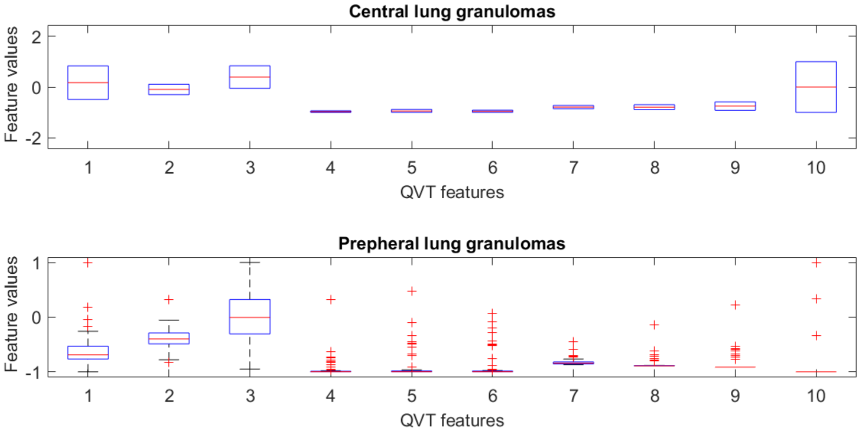

Figure S12. 3 QVT features (1,2: *Mean*, *Std* values of the vessel branches' torsion, 10: histogram of torsion) were significantly different between granulomas located within the central and peripheral regions of the lung.

G. Sensitivity of QVT features to errors in vasculature segmentation:

This section presents the sensitivity of QVT features to minor changes/ errors of the vasculature segmentation. We identified the parameter *S* which controls the growing of the vasculature volume during the segmentation process. This parameter corresponds to the mean difference value in Hounsfield units between a growing volume and the candidate voxels in the neighborhood of a vasculature. The values of *S* were identified empirically and belong to the following set: $S \in \{-150, -100, -50, 0, 50\}$. For each of the values in *S*, we generated multiple segmentations for each nodule and its surrounding vasculature on the CT scan. Fig.S13 (b,c,d,e,f) illustrates 5 segmentations of a nodule and its corresponding vasculature shown in Fig.S13 (a). Segmentations were generated based on the values of *S*.

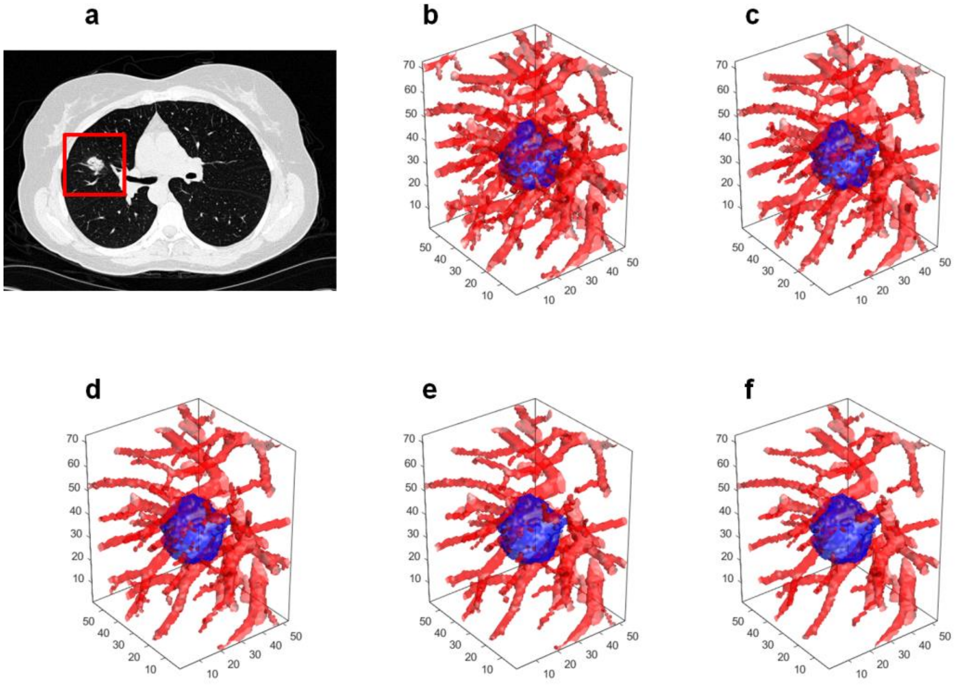

Fig S13. (a) A nodule and the region of interest. (b,c,d,e,f) Segmentations of the same nodule and its corresponding vasculature which were generated by assigning the following values respectively to the parameter *S*: -150, -100,-50, 0 and 50.

Then the QVT features were calculated for all 5 versions of segmentations. The QVT features were calculated for all of the training set examples. We denote the QVT features corresponding to each of the S values as QVT_{-150} , QVT_{-100} , QVT_{-50} , QVT_0 and QVT_{50} respectively. To measure sensitivity of QVT features to slight changes in vessel segmentation, we computed the correlation between QVT pairs which were denoted by $Corr(QVT_{s1}, QVT_{s2})$. Fig.S14 shows the 4 bar plots in which Fig.S13 (a,b,c,d) respectively illustrate $Corr(QVT_{-150}, QVT_{-100})$,

$Corr(QVT_{-100}, QVT_{-50})$, $Corr(QVT_{-50}, QVT_0)$ and $Corr(QVT_0, QVT_{50})$. As it may be appreciated from the bar plots, some features (13,14, 15, 16) were highly stable and correlated across changes in vessel segmentation. However, some features (17,18,19,20) were found to show more abrupt changes on account of minor perturbations in vessel segmentation. Consequently, as shown in the panels, these features were not highly correlated between different segmentations.

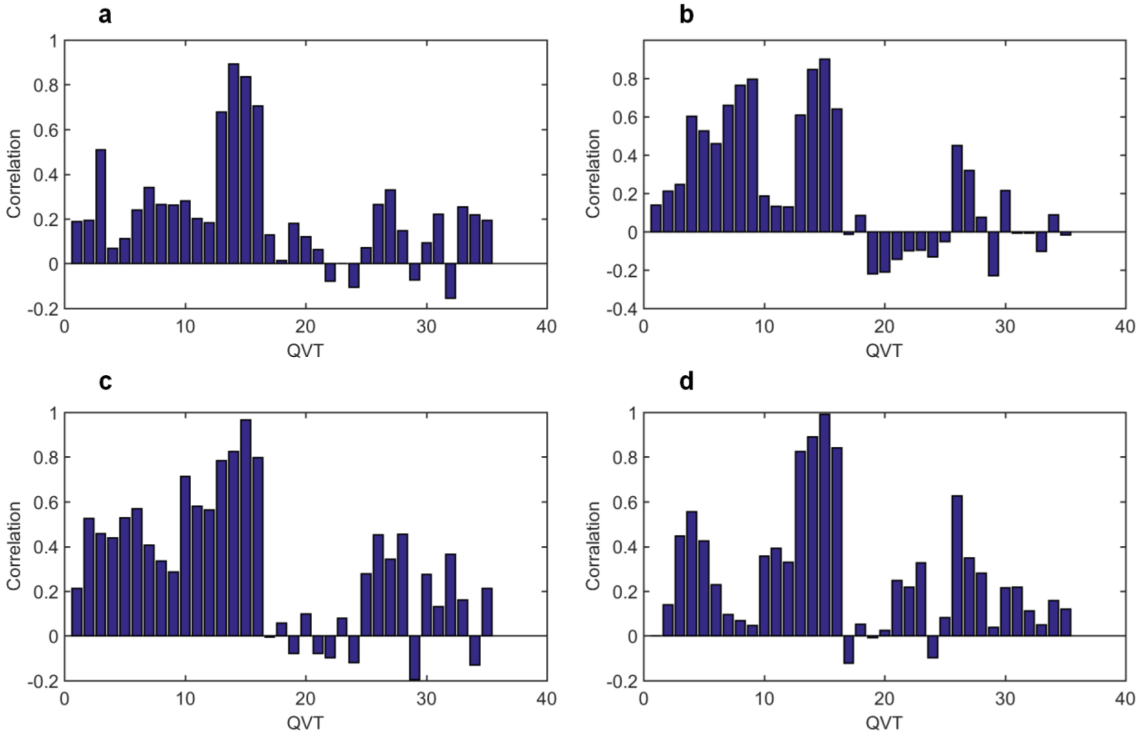

Fig S14. (a,b,c,d) respectively illustrate $\boldsymbol{Corr(QVT_{-150}, QVT_{-100})}$, $\boldsymbol{Corr(QVT_{-100}, QVT_{-50})}$, $\boldsymbol{Corr(QVT_{-50}, QVT_0)}$ and $\boldsymbol{Corr(QVT_0, QVT_{50})}$. As it may be appreciated from the bar plots, some features (13,14, 15, 16) were highly stable and correlated across changes in vessel segmentation. Specially, in panel (c) which corresponds to $\boldsymbol{Corr(QVT_{-100}, QVT_{-50})}$ most of the QVT features have shown high or moderate correlation. However, some features (17,18,19,20) were found to show more abrupt changes on account of minor perturbations in vessel segmentation. Consequently, as shown in the panels, these features were not highly correlated between different segmentations.

Additionally, we also computed classification performance of the $\boldsymbol{C_{QVT}}$ classifier as a function of $\boldsymbol{QVT_S}$. Fig.S15 and Table.S7 show the AUC values of the classifier for each $\boldsymbol{QVT_S}$, $\boldsymbol{S \in \{-150, -100, -50, 0, 50\}}$ **.**

Table S7. The AUC values of the classifier for each $\boldsymbol{QVT_S}$

Features	QVT_{-150}	QVT_{-100}	QVT_{-50}	QVT_0	QVT_{50}
AUC	0.79±0.06	0.82±0.12	0.87±0.11	0.75±0.14	0.69±18

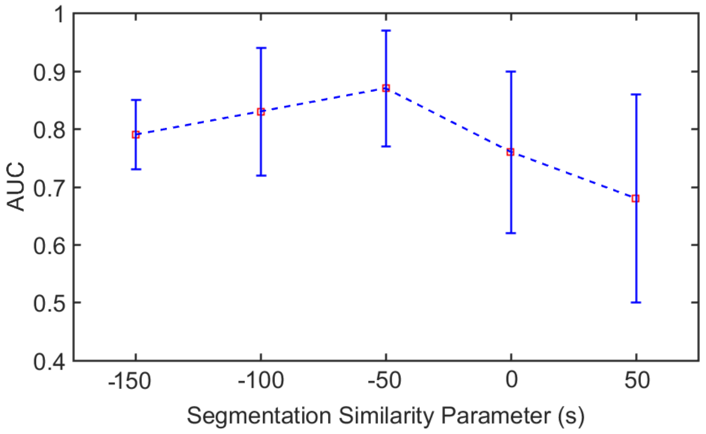

Fig S15. The AUC values of the C_{QVT} classifier for each QVT_S. The classifier yielded AUC= 0.79±0.06, 0.82±0.12, 0.87±0.11, 0.75±0.14 and 0.69±18 for respectively S=-150,-100, -50, 0 and 50. The AUC drops slightly as S changes toward its determined limits.

H. The effect of scanner variability and voxel size and smoking history on the QVT features:

The effect of scanner variability and voxel size on the QVT features. Fig.S16 presents the distribution of voxel size in both training and validation sets.

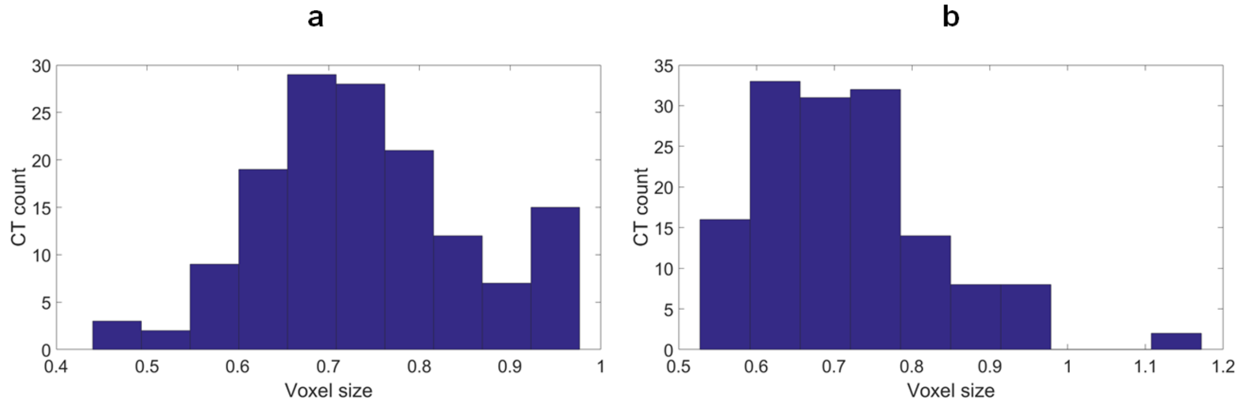

Figure S16. The distribution of voxel size (in millimeters) of the (a) training and (b) validation sets.

The statistical significance test result of voxel size and disease outcome is presented in Table S8.

Table S8. Statistical significance test between voxel size and disease outcome for both training and validation cohorts. The p-values were computed using two tailed and unpaired Students t test. P < 0.01 was considered as statistically significant.

Characteristic	*Training Set*				*Validation Set*			
	Adeno	*Grano*	*Total*	*p-value*	*Adeno*	*Grano*	*Total*	*p-value*
Voxel Size				**0.65**				**0.05**
Mean	0.73	0.74	0.74		0.70	0.74	0.72	
Std	0.11	0.11	0.11		0.11	0.12	0.12	
Median	0.72	0.73	0.72		0.68	0.72	0.70	

Additionally, we divided validation set into two sub-groups based on the voxel size (VS). There were 73 cases with VS>0.7 mm and 0.72 cases with VS<=0.7 mm. The AUC performance of the tortuosity based classifier in these was found to be 0.7 for VS<=0.7 and 0.81 for VS>0.7. Table S9 below provides details regarding the vendor and voxel size effect on the QVT classifier.

Table S9. The effect of vendor and voxel size on the QVT classifier The QVT classifier was trained with N=145 cases of the training set and tested on validation set’s subgroups created based on vendor and voxel size.

Criteria		*# of studies*	*AUC*
Vendor	Siemens	85	0.82
	Philips	58	0.72
Voxel size	VS>0.7 mm	73	0.81
	VS<=0.7	72	0.70

To show the impact of smoking history on the vessel architecture, we used a stratified sub set including 21 never smoker patients and 22 patients who had had at least 20 pack years. This stratified set include 8 never smokers and 9 smokers in Adenocarcinoma class. The Granuloma class included 13 never smokers and 13 smokers. QVT features were extracted for this subset and the QVT classifier validated on it. Table S10 shows details of the stratified sub set and AUC performance of the QVT based classifier on this dataset.

Table S10. Stratified sub set and AUC performance of the QVT based classifier this data set.

Status	*# of Adenocarcinoma*	*# of Granuloma*	*AUC*
Never smoked	8	13	0.73
Smoker	9	13	0.68
All	17	26	0.72

Additionally, the p-values of the null hypothesis that the difference of 5 selected QVT features between “Never smoked” and “Smoker” groups were found to be 0.61, 0.17, 0.03, 0.41, 0.65. In other words, no statistically significant difference were found for 4 out of 5 selected QVT features among “Never smoked” and “Smoker” groups .

I. The performance metric of the deep CNN across the training runs:

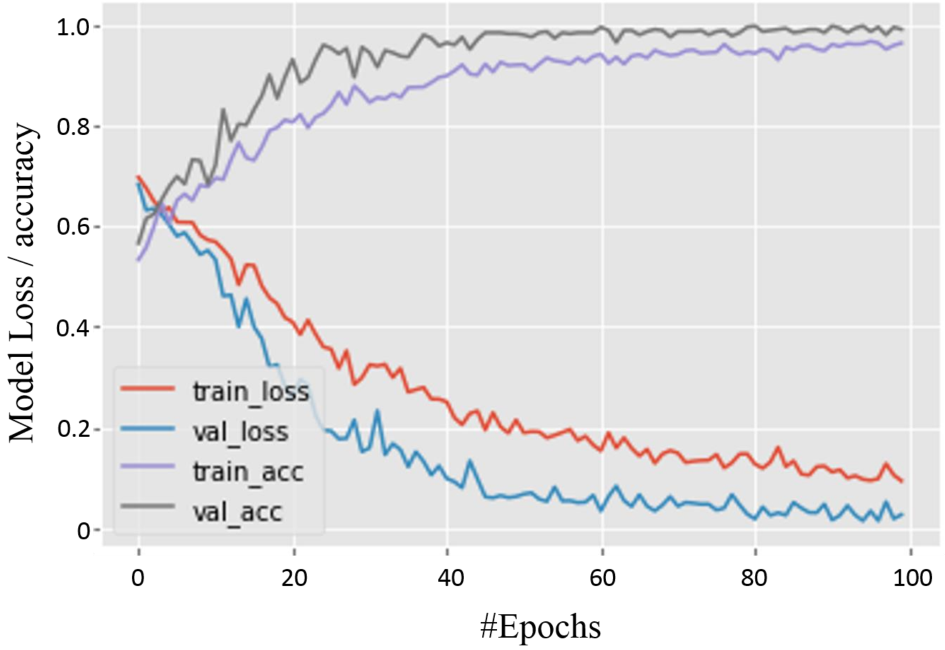

Figure S17. Training and Validation metrics across 100 epochs for the CNN. This plot shows that the model loss decreases over the epochs, and approaches zero. Similarly, model accuracy approaches 1 as number of epochs increases, and plateaus over 80 epochs.

J. Multi-fold cross validation on the entire dataset:

In this section, multi-fold cross validation was performed on the entire dataset, rather than just on the training set, to determine whether there was a difference in the selected features and the performance of the classifier. In this regard, we employed a 3-fold cross validation scheme (repeated 200 times) on the entire dataset which yielded an AUC=0.82±0.05. The corresponding ROC is shown in Fig.S18. Feature selection was performed within each iteration of the cross validation process and during each of the 600 iterations, we recorded the number of times that a feature was ranked among top three. For each QVT, Fig.S19 shows the number of times a feature was identified as being in the top 3 across multiple folds of cross-validation. As illustrated in Fig.S19, the features which were selected more than 100 times within the top ranked features, correspond to the top 12 features which were selected by our method and reported in the main manuscript. (there were 7 joint features between the feature selection strategies which was performed on training set only or on the entire dataset).

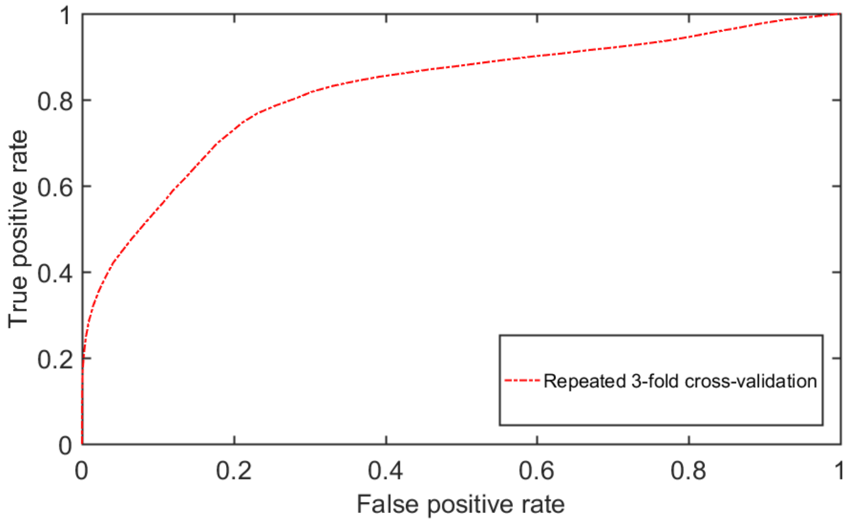

Figure S18. 3-fold cross validation across the entire dataset yielded an AUC=0.82±0.05

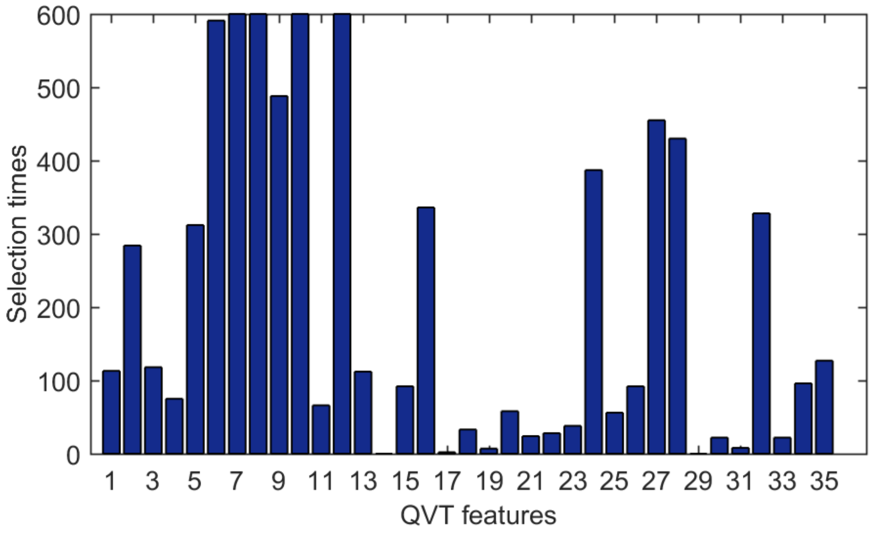

Figure S19.A frequency plot showing the number of times a QVT feature was identified as being within the top 3 ranked features across multiple runs of the cross-validation procedure**.**
